# The Biomechanics and Applications of Strongman Exercises: a Systematic Review

**DOI:** 10.1186/s40798-019-0222-z

**Published:** 2019-12-09

**Authors:** Benjamin R. Hindle, Anna Lorimer, Paul Winwood, Justin W. L. Keogh

**Affiliations:** 1 Faculty of Health Sciences and Medicine, Bond University, Gold Coast, Australia; 2 Department of Sport and Fitness, Faculty of Community Wellbeing and Development, Toi Ohomai Institute of Technology, Tauranga, New Zealand; 3 Sports Performance Research Institute New Zealand (SPRINZ), AUT Millennium Institute, AUT University, Auckland, New Zealand; 4 Kasturba Medical College, Manipal Academy of Higher Education, Mangalore, Karnataka India; 5 Cluster for Health Improvement, Faculty of Science, Health, Education and Engineering, University of the Sunshine Coast, Sunshine Coast, Australia

**Keywords:** Weightlifting, Kinematics, Kinetics, Motion analysis

## Abstract

**Background:**

The sport of strongman is becoming increasingly popular, catering for females, lightweight, and Masters competitors, with strongman exercises also being used by strength and conditioning coaches for a range of athletic groups. Thus, a systematic review was conducted to examine researchers’ current understanding of the biomechanics of strongman exercises, with a view to improve strongman athlete performance, provide biomechanical evidence supporting the transferability of strongman exercises to strength and conditioning/rehabilitation programs, and identify gaps in the current knowledge of the biomechanics of strongman exercises.

**Methods:**

A two-level search term strategy was used to search five databases for studies relevant to strongman exercises and biomechanics.

**Results:**

Eleven articles adherent to the inclusion criteria were returned from the search. The studies provided preliminary biomechanical analysis of various strongman exercises including the key biomechanical performance determinants of the farmer’s walk, heavy sled pull, and tire flip. Higher performing athletes in the farmer’s walk and heavy sled pull were characterized by a greater stride length and stride rate and reduced ground contact time, while higher performing athletes in the tire flip were characterized by a reduced second pull phase time when compared with lower performing athletes. Qualitative comparison of carrying/walking, pulling and static lifting strongman, traditional weight training exercises (TWTE), and common everyday activities (CEA), like loaded carriage and resisted sprinting, were discussed to further researchers’ understanding of the determinants of various strongman exercises and their applications to strength and conditioning practice. A lack of basic quantitative biomechanical data of the yoke walk, unilateral load carriage, vehicle pull, atlas stone lift and tire flip, and biomechanical performance determinants of the log lift were identified.

**Conclusions:**

This review has demonstrated the likely applicability and benefit of current and future strongman exercise biomechanics research to strongman athletes and coaches, strength and conditioning coaches considering using strongman exercises in a training program, and tactical operators (e.g., military, army) and other manual labor occupations. Future research may provide a greater understanding of the biomechanical determinants of performance, potential training adaptations, and risks expected when performing and/or incorporating strongman exercises into strength and conditioning or injury rehabilitation programs.

## Key Points

- Athletes with a greater overall performance outcome in the farmer’s walk and heavy sled pull could be biomechanically characterized by a greater stride length and stride rate and reduced ground contact time, while greater performance in the tire flip could be biomechanically characterized by a reduced second pull phase time.
- Biomechanical similarities were identified and discussed between the strongman farmer’s walk and yoke walk, and loaded backpack carriage; the strongman vehicle pull, and heavy sled pull and sub-body mass sled pull; and the strongman atlas stone lift, log lift and tire flip, and various phases of the clean and jerk, squat and deadlift.
- The existing literature demonstrated a lack of basic quantitative biomechanical data of the yoke walk, unilateral load carriage, vehicle pull, atlas stone lift and tire flip, and biomechanical performance determinants of the log lift.

## Background

Humankind’s obsession with strength dates back to antiquity, where wrestling matches were used to prove strength by the Greeks and Egyptians. To gain the strength, endurance, and power that were required to defeat their opponent, men would train by lifting stones of varying size, mass, and shape [1]. Around the twelfth century in Scotland, the Highland Games became popular for determining the strongest competitor, with competitors required to perform running, jumping, lifting and wrestling tasks to prove their strength. Contemporary Highland Games have evolved to include heavy throwing and lifting events. The increasing popularity and international awareness of the Highland Games throughout the twentieth century led to “The World’s Strongest Man”, first held in 1977 [2, 3]. In recent years, the sport of strongman has undergone rapid growth with competitions at local, regional, national and international levels, and a range of divisions created to cater for age, body mass, gender, and experience [4].

**Fig. 1:**
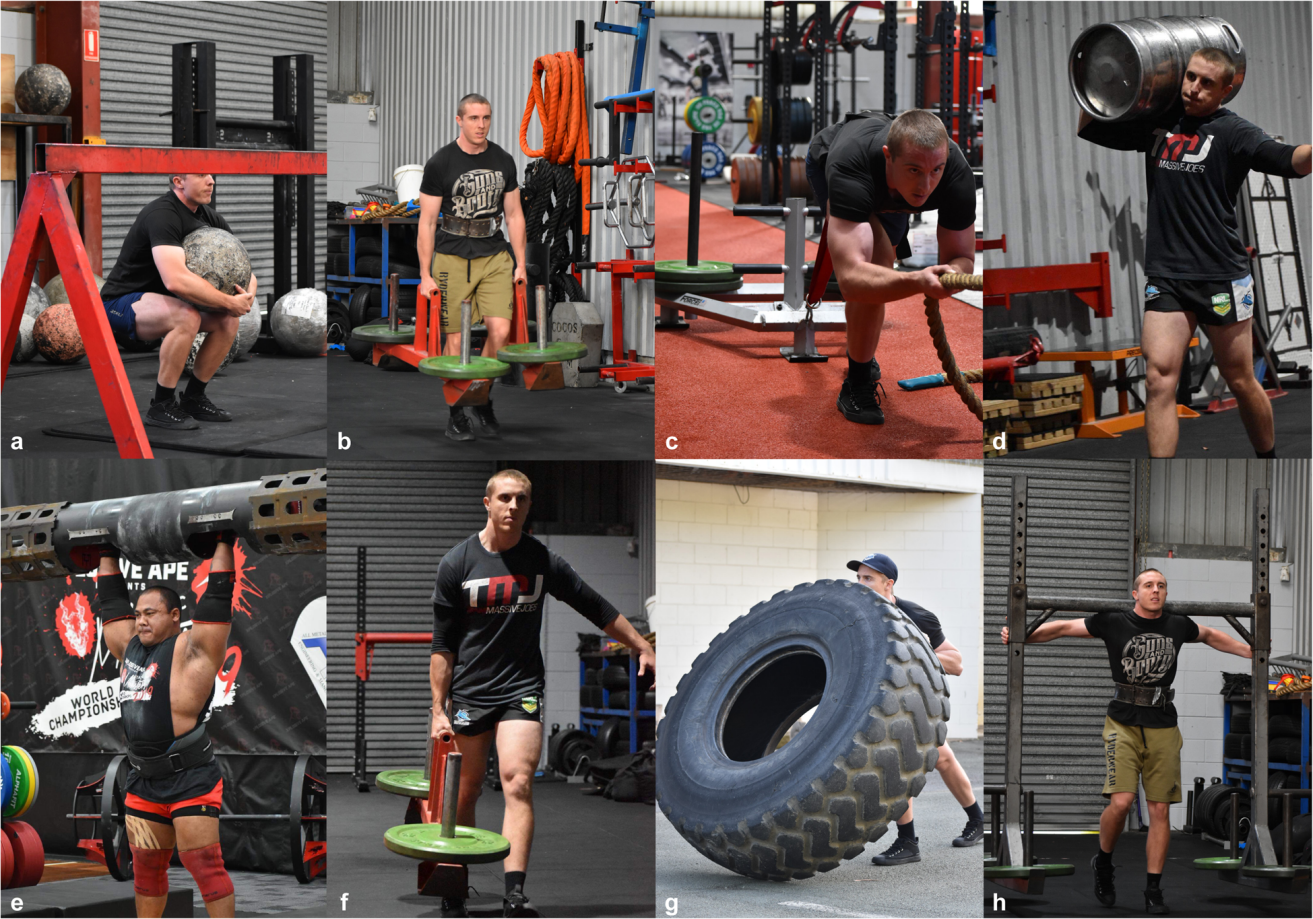
Examples of strongman exercises: **a** atlas stone lift, **b** farmer’s walk, **c** heavy sled pull, **d** keg walk, **e** log lift, **f** suitcase carry, **g** tire flip, **h** yoke walk. Images reprinted with permission from owner Hiroya Togawa

Modern strongman competitions require an athlete to carry, pull or lift heavy and awkward objects [5]. The exercises developed for strongman competitions are generally heavier versions of common everyday activities (CEA), or more awkward/challenging variations of traditional weight training exercises (TWTE) such as the squat, deadlift, and clean and jerk [6]. In contrast to TWTE, which typically require the weight to be lifted vertically and use bilateral load distribution, strongman exercises often require the athlete to move loads horizontally, test the athlete in multiple planes and incorporate phases of unilateral and bilateral loading [7, 8]. Strongman exercises typically utilize equipment such as loaded frames, kegs and bags for carrying, loaded sleds, and vehicles for pulling, as well as stones, logs, tires and oversized dumbbells for lifting (Fig. 1) [9].

As the sport of strongman continues to increase in popularity, and the use of such exercises in strength and conditioning programs becomes more common for non-strongman athletes, research in this area continues to grow. Strongman research has largely been an investigation of the use of strongman implements by strength and conditioning coaches of non-strongman athletes [10], the acute and chronic physiological adaptations to strongman-type training [5, 6, 11–14], the training and tapering practices of strongman athletes [2, 15–19], and the injury epidemiology of strongman athletes [4]. The literature now also includes a systematic review of the biomechanical research methods used in strongman studies [20], and narrative reviews and opinion pieces suggesting how strongman exercises may be best used in the strength and conditioning programs of non-strongman athletes [2, 14, 15, 19].

As the authors explored the current field of strongman biomechanics research, it became apparent that to thoroughly report and discuss all data on the biomechanics of strongman exercises a second systematic review further to Hindle et al. [20] was required. The first strongman biomechanics systematic review focused on the research methods used in existing strongman biomechanics research, whereby a summary of the exercises, study designs, study populations and biomechanical methods/measurements used were reported [20].

The primary objective of the current systematic review was to determine our understanding of the biomechanics of strongman exercises specifically with the following views: (1) improve athlete performance by providing athletes and coaches with a greater understanding of the key biomechanical determinants of performance of these exercises; (2) provide biomechanical evidence supporting the transferability of strongman exercises to the strength and conditioning/rehabilitation programs of athletes, tactical operators (e.g., military, army), and other manual labor occupations; and (3) identify the gaps in the current knowledge of the biomechanics of strongman exercises. Such information would be valuable to the strongman coach and athlete, the strength and conditioning coach who may use these exercises with their non-strongman athletes, as well as the researcher who may design future studies to address some of the key limitations of the literature.

## Methods

### Experimental Approach to the Problem

The review process followed the “Preferred Reporting Items for Systematic Reviews and Meta-Analyses” (PRISMA) guidelines on reporting items for a systematic review and the associated PRISMA checklist [21]. Due to the nature of the systematic review, Institutional Review Board approval to conduct this investigation and registration with the International Prospective Register of Systematic Reviews (PROSPERO) was not deemed to be relevant. A set of inclusion/exclusion criteria were developed prior to undertaking the search process. The criteria specified only peer-reviewed journal articles assessing anthropometric, kinematic, kinetic, muscular activity, or spatiotemporal measures of athletes performing common strongman exercises would be included in the review. Articles including injured athletes, sled loads less than the body mass of the athlete, and studies which focused on the use of the sled pull for the purpose of sprint performance would be excluded from the primary literature reviewed. No limitations were placed on language or year of publication. The data from the included articles were then extracted, analyzed and discussed based on the strongman exercise type.

### Literature Search and Screening

To identify all articles in which biomechanical analysis of a strongman exercise had been undertaken, a two-level keyword search consisting of terms associated with strongman exercises, lifts and training methods (level one), and terms associated with general biomechanical parameters (level two) was constructed using Boolean operators. An initial search up to and including 25 October 2018 was conducted using AusportMed, CINAHL, Embase, Medline (Ovid), and SPORTDiscus. The search was repeated on 25 March 2019 so to identify any articles published since the initial search. The full search strategy used for each database can be observed in Additional file 1: Table S1.

The results from the five databases were imported into online systematic review management software Rayyan (Doha, Qatar) before being distributed to two independent reviewers [22]. The two reviewers cast either “include,” “exclude,” or “maybe” votes for each article throughout the title/abstract screening process and “include” or “exclude” votes during the full text screening process in accordance with the predefined inclusion/exclusion criteria. During the full text screening process, reviewers were required to provide reasons based on a list of hierarchical criteria as to why they were excluding a study from further review. Where disagreement in voting or reasoning for exclusion occurred, a consensus meeting was held to form an agreement between parties. After identifying all eligible articles, the reference list of each article was examined, and Google Scholar was used to perform a forward citation search to identify any potentially eligible articles not returned during the database search.

### Risk of Bias and Quality Assessment

No single risk of bias/quality assessment tool appeared entirely suitable to perform a meaningful assessment of the identified literature, which were all of a cross-sectional observational study design. An appropriate checklist was developed by the authors using tools established by other systematic reviews containing studies of a similar design [23–30], with this adapted checklist used by Hindle et al. [20] in a systematic review of the biomechanical research methods used to evaluate strongman exercises. Reviewers awarded a star (☼) in support of a criterion, or no star where a criterion was not met, with any disagreement in voting between reviewers settled by a consensus meeting. The risk of bias score was calculated for each article based on a total maximum achievable score of 16 stars, and categorized in accordance with Davids et al. [24], where articles scoring ≥ 11 stars were categorized as having a low risk of bias, articles scoring 6–10 stars categorized as having a satisfactory risk of bias, and articles scoring ≤ 5 stars categorized as having a high risk of bias.

## Results

### Literature Search and Screening

**Fig. 2:**
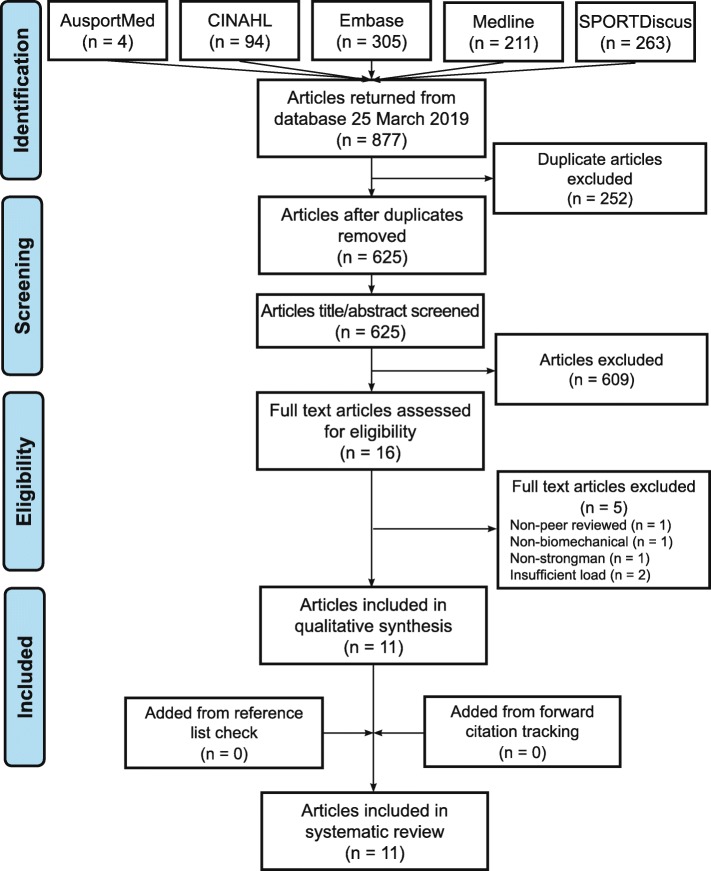
Flowchart of the screening process

The search of the five databases on 25 March 2019 returned 877 results, of which eleven articles were identified as being adherent to the inclusion criteria (Fig. 2). The outcome of the screening process and resultant PRISMA flowchart differed slightly to Hindle et al. [20] as the independent reviewers agreed upon excluding a greater number of articles at the title/abstract level due to familiarization with these articles during previous full text screening.

### Risk of Bias and Quality Assessment

All studies clearly stated the objectives/purpose of the study, the characteristics of the study population, and used appropriate statistical methods during data analysis. The majority of studies also proposed a testable hypothesis and used well-validated equipment to collect measures. All articles were classified as having a satisfactory or low risk of bias (Table 1).

**Table 1:** Risk of bias and quality assessment

Article	1	2	3	4	5	6	7	8	9	10	11	12	13	14	15	16	Score (/16)
Holmstrup et al. [31]	-	☼	☼	☼	☼	-	-	☼	-	☼	☼	☼	☼	☼	☼	☼	12 (L)
Keogh et al. [32]	☼	☼	☼	-	☼	-	☼	☼	☼	☼	☼	☼	☼	-	☼	☼	13 (L)
Keogh et al. [33]	☼	☼	☼	-	☼	-	-	☼	☼	☼	☼	☼	☼	-	☼	☼	11 (L)
Keogh et al. [34]	☼	☼	☼	-	☼	-	-	☼	☼	☼	☼	☼	☼	☼	☼	☼	13 (L)
McGill et al. [35]	-	☼	☼	-	☼	☼	-	-	-	-	☼	☼	☼	-	-	-	7 (S)
Stastny et al. [36]	☼	☼	-	-	☼	-	-	☼	-	☼	☼	-	-	-	☼	☼	8 (S)
Renals et al. [37]	☼	☼	☼	-	☼	☼	-	☼	☼	☼	☼	☼	☼	-	-	☼	12 (L)
Winwood et al. [8]	☼	☼	☼	-	☼	-	-	☼	☼	☼	☼	☼	☼	-	-	☼	11 (L)
Winwood et al. [7]	☼	☼	☼	☼	☼	☼	-	☼	☼	☼	☼	☼	☼	☼	-	-	13 (L)
Winwood et al. [38]	☼	☼	☼	☼	☼	☼	-	☼	☼	☼	☼	☼	☼	☼	-	-	13 (L)
Winwood et al. [39]	☼	☼	☼	☼	☼	☼	-	☼	☼	☼	☼	☼	☼	-	-	-	12 (L)

Method for assessing risk of bias: **(1)** study design was stated clearly; **(2)** the study objective/purpose is clearly stated; **(3)** the study has a clearly testable hypothesis; **(4)** the study clearly states the inclusion criteria for participants; **(5)** the characteristics of the population are well detailed; **(6)** the study population is representative of the intended population for which the research is aimed; **(7)** a justification for the selection of the sample/study population size was provided; **(8)** the methods used throughout testing are well detailed; **(9)** the measurement tools used throughout the study are reliable and have been validated; **(10)** detail on the statistical methods used was provided; **(11)** the statistical methods used to analyze the data were appropriate; **(12)** the results of the study are well detailed; **(13)** the information provided in the paper is sufficient information was provided so to allow the reader to make an unbiased assessment of the study findings; **(14)** confounding factors within the study are identified; **(15)** study funding/conflicts of interest were acknowledged; **(16)** limitations to the study were identified. *L* low risk of bias (11–16 ☼), *S* satisfactory risk of bias (6–10 ☼), *H* high risk of bias (0–5 ☼)

### Study Results and Data Synthesis

**Fig. 3:**
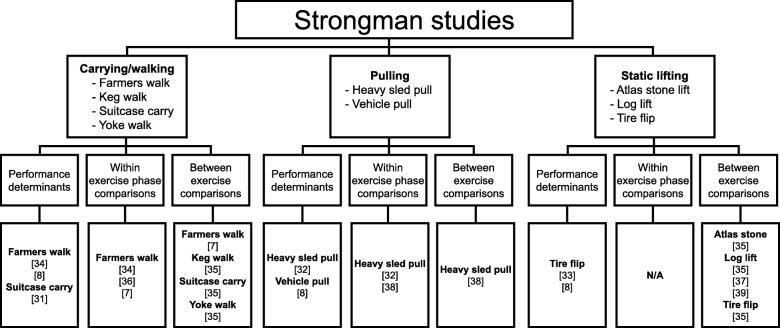
Study results structure

Strongman exercises which have had a biomechanical assessment in at least one of the eleven studies were the atlas stone lift, farmer’s walk, heavy sled/vehicle pull, log lift, keg walk, suitcase carry, tire flip, and yoke walk (Fig. 1). For a description of these exercises, the reader is directed to Hindle et al. [20]. The eight strongman exercises could be categorized into three exercise types: carrying/walking, pulling, and static lifting (Fig. 3). The comparative analysis within each of the studies could be categorized into three main areas: comparisons based on the performance outcome of the exercise [8, 31–34], within exercise comparisons (between phase) [7, 32, 34, 36, 38], and between exercise comparisons [7, 35, 37–39]. The results from the eleven studies are presented in the format outlined in Fig. 3.

### Carrying/Walking Exercises

The carrying/walking strongman exercises biomechanically analyzed were the farmer’s walk, keg walk, suitcase carry, and yoke walk. The farmer’s walk was the most studied exercise, enabling within and between study comparisons (Table 2).

**Table 2:** Walking/carrying results comparisons—farmer’s walk

	Winwood et al. [7]			Keogh et al. [34]			
	Farmer’s walk	Unloaded walk	Effect size	Higher performer	Lower performer	Effect size	Group ave.
Spatiotemporal							
Ground contact time (s)	0.46 ± 0.06* (MVP)	0.67 ± 0.06 (MVP)	− 3.50	0.29 ± 0.02^†^ (MVP^˟^)	0.34 ± 0.03 (SVP^˟^)	− 1.96	0.30 ± 0.03^☼^ (MVP)
	0.53 ± 0.09*(AP)	0.77 ± 0.07 (AP)	− 3.00	0.39 ± 0.04^†^ (AP)	0.32 ± 0.03 (AP)	1.98	0.36 ± 0.04 (AP)
Stride rate (Hz)	1.42 ± 0.17* (MVP)	0.88 ± 0.06 (MVP)	4.20	2.01 ± 0.13^†^ (MVP^˟^)	1.83 ± 0.04 (SVP^˟^)	1.88	1.97 ± 0.13^☼^ (MVP)
	1.21 ± 0.12* (AP)	0.82 ± 0.04 (AP)	4.40	1.88 ± 0.10^†^ (AP)	1.64 ± 0.12 (AP)	2.17	1.79 ± 0.14 (AP)
Stride length (m)	1.04 ± 0.12* (MVP)	1.43 ± 0.11 (MVP)	− 3.40	1.83 ± 0.04^†^ (MVP^˟^)	1.40 ± 0.17 (SVP^˟^)	3.48	1.67 ± 0.10^☼^ (MVP)
	0.85 ± 0.19* (AP)	1.33 ± 0.11 (AP)	− 3.10	1.38 ± 0.16 (AP)	1.33 ± 0.09 (AP)	0.39	1.32 ± 0.12 (AP)
Average velocity (m/s)	1.48 ± 0.19 (MVP)	1.26 ± 0.15 (MVP)	1.28	3.66 ± 0.17^†^ (MVP)	2.83 ± 0.36 (MVP)	2.95	3.29 ± 0.38^☼^ (MVP)
	1.05 ± 0.21 (AP)	1.11 ± 0.09 (AP)	− 0.37	2.61 ± 0.38 (AP)	2.19 ± 0.27 (AP)	1.27	2.41 ± 0.32 (AP)
Kinematic							
Ankle angle at FS (°)	95.0 ± 3.00* (MVP)	105 ± 2.00 (MVP)	− 3.80	101 ± 6.00^†^ (SVP^˟^)	113 ± 5.00 (MVP^˟^)	− 2.17	110 ± 9.00 (MVP)
	96.00 ± 6.00* (AP)	105 ± 2.00 (AP)	− 2.30	99.0 ± 8.00 (AP)	106 ± 6.00 (AP)	− 0.99	100 ± 8.00 (AP)
Ankle angle at TO (°)	100 ± 5.00* (MVP)	115 ± 9.00 (MVP)	− 2.10	118 ± 5.00 (MVP^˟^)	117 ± 7.00 (SVP^˟^)	0.16	114 ± 6.00(MVP)
	105 ± 6.00 (AP)	118 ± 5.00 (AP)	− 2.30	108 ± 4.00^†^ (AP)	114 ± 3.00 (AP)	− 1.70	111 ± 5.00 (AP)
Ankle ROM (°)	4.00 ± 4.00 (MVP)	10.0 ± 10.0 (MVP)	− 0.70	− 10.0 ± 4.00^†^ (SVP)	1.00 ± 5.00 (MVP)	− 2.43	− 4.00 ± 7.00^☼^ (MVP)
Knee angle at FS (°)	154 ± 7.00* (MVP)	178 ± 6.00 (MVP)	− 3.70	156 ± 6.00 (MVP^˟^)	166 ± 16.0 (SVP^˟^)	− 0.83	155 ± 6.00^☼^ (MVP)
	150 ± 9.00* (AP)	174 ± 10.0 (AP)	− 2.50	147 ± 7.00 (AP)	151 ± 5.00 (AP)	− 0.66	150 ± 6.00 (AP)
Thigh angle at FS (°)	34.0 ± 6.00* (MVP)	23.0 ± 7.00 (MVP)	1.80	38.0 ± 3.00^†^ (MVP^˟^)	31.0 ± 4.00 (SVP^˟^)	1.98	34.0 ± 3.00 (MVP)
Thigh ROM (°)	− 19.0 ± 5.00 (MVP)	− 22.0 ± 10.0 (MVP)	0.40	− 44.0 ± 4.00^†^ (MVP^˟^)	− 35.0 ± 6.00 (SVP^˟^)	− 1.77	− 38.0 ± 4.00^☼^ (MVP)
Trunk angle at FS (°)	78.0 ± 3.00* (MVP)	90.0 ± 2.00 (MVP)	− 4.10	–	–		–
	69.0 ± 5.00* (AP)	85.0 ± 2.00 (AP)	− 4.30	–	–		–
Trunk angle at TO (°)	76.0 ± 4.00* (MVP)	87.0 ± 2.00 (MVP)	− 3.20	–	–		–
	70.0 ± 5.00* (AP)	84.0 ± 4.00 (AP)	− 3.40	–	–		–
Kinetic							
Mean anterior GRF (N)	127 ± 31.0* (MVP)	83.0 ± 25.0 (MVP)	1.60	–	–		–
Peak anterior GRF (N)	447 ± 98.0* (MVP)	259 ± 53.0 (MVP)	2.40	–	–		–
Mean medial GRF (N)	120 ± 41.0* (MVP)	70.0 ± 36.0 (MVP)	1.30	–	–		–
Peak medial GRF (N)	241 ± 73.0* (MVP)	120 ± 62.0 (MVP)	1.80	–	–		–
Mean posterior GRF (N)	159 ± 45.0* (MVP)	94.0 ± 34.0 (MVP)	1.60	–	–		–
Peak posterior GRF (N)	389 ± 143* (MVP)	211 ± 77.0 (MVP)	1.50	–	–		–
Mean vertical GRF (N)	2540 ± 376* (MVP)	1030 ± 247 (MVP)	4.70	–	–		–
Peak vertical GRF (N)	3630 ± 608* (MVP)	1510 ± 387 (MVP)	4.10	–	–		–
Peak lateral GRF (N)	210 ± 73.0* (MVP)	119 ± 45.0 (MVP)	1.50	–	–		–

All data are reported as means ± standard deviation, unless specified otherwise. Effect sizes reported for between exercise [7] and performance standard [31]. A positive effect size indicates that the left-hand column (farmer’s walk or higher performer) had a greater value than the respective right-hand column (unloaded walk or lower performer). ^*^Significant difference to unloaded walking; ^†^significant difference to low performing athletes, ^☼^significant difference to acceleration phase, ^˟^comparison between phase based on distance, *AP* acceleration phase, *ave* average, *GRF* ground reaction force, *MVP* maximum velocity phase, *SVP* sub-maximal velocity phase

#### Biomechanical Determinants of Performance

Comparing spatiotemporal measures of higher performing (HP) with lower performing (LP) athletes, greater performance in the farmer’s walk was associated with a reduced ground contact time, increased stride length, and increased stride rate during the maximum velocity phase of the walk [34]. Maximum velocity was reached at different stages of the farmer’s walk depending on the athlete’s performance level, with HP athletes reaching a maximum velocity in the final 17–20 m section of the walk, while LP athletes reached a maximum velocity in the middle 8.5–11.5 m section of the walk [34].

HP athletes exhibited statistically greater dorsiflexion of the ankle at foot strike and toe off, a more horizontally aligned thigh at foot strike, and greater ankle and thigh range of motion (ROM) [34]. Measures of flexed arm girth, muscle mass and total system force (calculated as the sum of the athlete’s body mass and one-repetition maximum (1RM) squat) were reported to be the greatest anthropometric determinants of performance in the farmer’s walk exercise (flexed arm girth, *r* = 0.46; muscle mass, *r* = 0.49; total system force, *r* = 0.64) [8]. Participants with a greater percentage of fat-free mass were also found to be able to carry greater loads during the suitcase carry before their technique and posture were compromised [31].

#### Within Exercise Biomechanical Differences

Reduced ground contact time and increased stride length were observed in the maximum velocity phase of the farmer’s walk when compared with the acceleration and sub-maximal velocity phase [7, 34]. Comparing acceleration, sub-maximal and maximal velocity phases, greater ankle dorsiflexion and knee flexion at foot strike, greater knee flexion and a more horizontally oriented thigh at toe off, increased ankle ROM, and reduced thigh and knee ROM were observed during the acceleration phase [34]. Stastny et al. [36] compared muscle activation patterns between athletes of varying muscle activation strength ratios, finding athletes with a hip abductor (HAB) to hamstring maximum voluntary contraction (MVC) ratio < 1 and/or a HAB to quad MVC ratio < 0.5, tended to have greater activation of the gluteus medius muscle during the farmer’s walk.

#### Between Exercise Biomechanical Differences

Statistically greater stride rate, and reduced stride length and ground contact time have been reported in the farmer’s walk when compared with unloaded walking [7]. The farmer’s walk was also observed to result in greater anterior tilt of the trunk, dorsiflexion of the ankle and extension of the knee at foot strike, and increased mean and peak anterior, posterior, vertical and medial ground reaction forces [7].

Comparison of joint/segment angular kinematics of the initial lift of the farmer’s walk (farmer’s lift) with the deadlift indicated the farmer’s lift to be primarily characterized by a more vertical trunk position throughout the majority of the lift except for at lift completion, leading to an overall reduced trunk ROM [7]. Greater mean vertical, anterior, and resultant anterior/posterior forces were also reported in the farmer’s lift [7]. The only reported differences in lumbar joint angular kinematics during the carrying/walking exercises were a greater peak twist angle during the right-hand suitcase carry (~ 11°) than the farmer’s walk (~ 8°) and yoke walk (~ 7°), which were all statistically greater than the left-hand suitcase carry (~ 6°) [35].

The farmer’s walk and yoke walk bilateral load carriage exercises were reported to result in statistically greater muscular compression, anterior/posterior spine muscular loading, muscular axial twist stiffness, and flexion/extension stiffness than the right/left-hand suitcase carry unilateral load carriage exercise [35]. McGill et al. [35] reported greater activation of a number of key spinal musculature when performing the yolk and farmer’s walk than when performing the left/right-hand suitcase carry. Further statistical differences in muscle activation patterns are presented in Table 3.

**Table 3:** Significant differences in muscle activation and kinetic outcomes between the walking/carrying exercises

	Farmer’s walk [35]	LH suitcase carry [35]	RH suitcase carry [35]	Yoke walk [35]
Muscle activity (%MVC)				
Left upper erector spinae	77.6 ± 29.3^‡^	47.1 ± 6.20	32.4 ± 4.60*^☼^	69.3 ± 17.5^‡^
Right upper erector spinae	91.4 ± 54.7^†^	24.9 ± 17.6*^‡☼^	52.1 ± 17.3^†^	65.6 ± 14.4^†^
Left lower erector spinae	106 ± 51.1^†^	31.6 ± 10.1*^‡☼^	77.4 ± 21.3^†^	79.2 ± 10.2^†^
Right lower erector spinae	144 ± 36.7^‡^	96.9 ± 20.4^‡^	44.1 ± 9.10*^†☼^	107 ± 31.5^‡^
Left latissimus dorsi	169 ± 55.4*^‡^	97.4 ± 55.7	68.9 ± 23.2^☼^	51.9 ± 26.4^☼^
Right latissimus dorsi	152 ± 26.7*^†^	65.3 ± 6.20^☼^	91.4 ± 39.1	45.5 ± 31.7^☼^
Left external oblique	39.3 ± 30.6^†^	12.6 ± 5.30*^‡☼^	61.5 ± 21.9^†^	47.5 ± 31.7^†^
Right external oblique	50.4 ± 17.4^‡^	65.1 ± 24.4^‡^	29.0 ± 17.8*^†☼^	58.8 ± 17.4^‡^
Right rectus abdominis	13.3 ± 3.80	14.6 ± 4.50	5.60 ± 1.80*	22.3 ± 18.1^‡^
Right gluteus maximus	114 ± 70.3^‡^	78.2 ± 39.5	50.5 ± 31.2*^☼^	113 ± 52.1^‡^
Right gluteus medius	108 ± 66.9^‡^	64.1 ± 38.7	57.3 ± 23.6*^☼^	108 ± 69.7^‡^
Right bicep femoris	54.0 ± 13.7^†^	31.2 ± 7.50*^‡☼^	48.3 ± 8.6^†^	61.7 ± 6.30^†^
Right rectus femoris	77.4 ± 35.6	41.1 ± 9.20*	56.5 ± 11.5*	107 ± 23.5^†‡^
Kinetic				
Muscular anterior/posterior shear (N)	2800^†^	1680^☼^	1160	1890
Muscular compressive load (N)	7900^†^	5800*^☼^	6700	7800^†^
Muscular axial twist stiffness (Nm/rad)	27,200^†‡^	19,100^☼^	24,600^☼^	25,900
Muscular flexion/extension stiffness (Nm/rad)	35,600	24,000*	27,500	38,600^†^

All data are reported as means ± standard deviation, unless specified otherwise. *Significant difference to yoke walk, ^†^significant difference to left-hand suitcase carry, ^‡^significant difference to right-hand suitcase carry, ^☼^significant difference to farmer’s walk, *LH* left hand, *MVC* maximum voluntary contraction, *RH* right hand

### Pulling Exercises

The only pulling strongman exercise biomechanically analyzed was the heavy sled pull, while anthropometric measures were assessed and correlated to performance in the truck/vehicle pull. Basic within and between study comparison of the heavy sled pull could be conducted using the available data (Table 4).

**Table 4:** Pulling significant results comparisons—heavy sled pull

	Winwood et al. [38]			Keogh et al. [32]			
	Heavy sled pull	Effect size	Back squat	Higher performer	Lower performer	Effect size	Group ave.
Spatiotemporal							
Ground contact time (s)	0.35 ± 0.04 (MVP)	− 0.85	–	0.33 ± 0.04^†^ (MVP)	0.76 ± 0.37 (MVP)	− 1.63	0.48 ± 0.23 (MVP)
	0.38 ± 0.03 (AP)		–	0.42 ± 0.19 (AP)	0.57 ± 0.23 (AP)	− 0.71	0.53 ± 0.32 (AP)
Stride rate (s)	1.42 ± 0.14 (MVP)	0.07	–	1.63 ± 0.12^†^ (MVP)	1.10 ± 0.42 (MVP)	1.72	1.37 ± 0.39 (MVP)
	1.41 ± 0.14 (AP)		–	1.50 ± 0.55 (AP)	1.29 ± 0.37 (AP)	0.45	1.45 ± 0.50 (AP)
Swing time (s)	0.33 ± 0.04 (MVP)	0.39	–	0.29 ± 0.03 (MVP)	0.27 ± 0.05 (MVP)	0.49	0.28 ± 0.04^☼^ (MVP)
	0.31 ± 0.06 (AP)		–	0.28 ± 0.07^†^ (AP)	0.23 ± 0.05 (AP)	0.82	0.25 ± 0.06 (AP)
Stride length (m)	1.29 ± 0.17^☼^ (MVP)	1.81	–	1.29 ± 0.26^†^ (MVP)	0.80 ± 0.16 (MVP)	2.27	1.03 ± 0.26^☼^ (MVP)
	1.00 ± 0.15 (AP)		–	0.85 ± 0.25^†^ (AP)	0.65 ± 0.04 (AP)	1.12	0.74 ± 0.28 (AP)
Kinematic							
Average velocity (m/s)	1.83 ± 0.22^☼^ (MVP)	2.44	–	2.08 ± 0.08^†^ (MVP)	0.99 ± 0.50 (MVP)	3.04	1.61 ± 0.55^☼^ (MVP)
	1.39 ± 0.13 (AP)		–	1.22 ± 0.20^†^ (AP)	0.79 ± 0.32 (AP)	1.61	1.04 ± 0.30 (AP)
Knee angle at FS (°)	114 ± 6.00^☼^ (MVP)	1.38	–	132 ± 9.00^†^ (MVP)	112 ± 22.0 (MVP)	1.19	124 ± 18.0^☼^ (MVP)
	103 ± 9.00(AP)		–	125 ± 12.0^†^ (AP)	110 ± 10.0(AP)	1.36	116 ± 13.0 (AP)
Knee angle at TO (°)	138 ± 14.0 (MVP)	0.35	–	153 ± 7.00 (MVP)	148 ± 10.0(MVP)	0.59	149 ± 9.00^☼^ (MVP)
	133 ± 14.0 (AP)		–	148 ± 14.0^†^ (AP)	138 ± 17.0 (AP)	0.65	141 ± 15.0 (AP)
Thigh angle at FS (°)	–	–	–	23.0 ± 5.00^†^ (MVP)	19.0 ± 5.0 (MVP)	0.80	21.0 ± 5.00^☼^ (MVP)
	–		–	14.0 ± 10.0 (AP)	16.0 ± 8.00 (AP)	− 0.22	15.0 ± 10.0 (AP)
Trunk angle at FS (°)	61.0 ± 13.0 (MVP)	− 0.67	–	41.0 ± 7.00^†^ (MVP)	8.00 ± 29.0 (MVP)	1.56	26.0 ± 24.0^☼^ (MVP)
	77.0 ± 30.0 (AP)		–	29.0 ± 17.0^†^ (AP)	2.00 ± 16.0 (AP)	1.64	14.0 ± 21.0 (AP)
Trunk angle at TO (°)	61.0 ± 11.0 (MVP)	− 0.49	–	41.0 ± 9.00^†^ (MVP)	14.0 ± 25.0 (MVP)	1.59	28.0 ± 21.0^☼^ (MVP)
	69.0 ± 20.0 (AP)		–	31.0 ± 15.0^†^ (AP)	10.0 ± 14.0 (AP)	1.45	19.0 ± 19.0 (AP)
Kinetic							
Mean anterior GRF (N)	555 ± 107* (SC to MKE)	6.63	43.0 ± 22.0 (SC to MKE)	–	–		–
Peak anterior GRF (N)	810 ± 174* (SC to MKE)	5.13	126 ± 73.0 (SC to MKE)	–	–		–
Mean vertical GRF (N)	1330 ± 364* (SC to MKE)	− 2.38	2580 ± 648 (SC to MKE)	–	–		–
Peak vertical GRF (N)	1740 ± 463* (SC to MKE)	− 1.85	3500 ± 1270 (SC to MKE)	–	–		–
Mean resultant ant/post force (N)	271 ± 89.0^☼^ (MVP)	− 1.95	–	–	–	–	–
	526 ± 162 (AP)		–	–	–	–	–
Mean resultant med/lat force (N)	− 5.00 ± 22.0^☼^ (MVP)	− 1.75	–	–	–	–	–
	24.0 ± 8.00 (AP)		–	–	–	–	–

All data are reported as means ± standard deviation, unless specified otherwise. Spatiotemporal and kinematic effect sizes reported for between phase [37] and between performance standard [32]. Kinetic effect sizes reported for between exercise (heavy sled pull vs back squat). A positive effect size indicates that the left-hand column (higher performer or heavy sled pull) or top row (maximum velocity phase) had a greater value than the respective right-hand column (lower performer or back squat) or bottom row (acceleration phase).*Significant difference to back squat, ^†^significant difference to low performing athletes, ^☼^^☼^significant difference to acceleration phase, *ant/post* anterior/posterior, *AP* acceleration phase, *ave* average, *FS* foot strike, *GRF* ground reaction force, *med/lat* medial/lateral, *MVP* maximum velocity phase, *SC to MKE* start of concentric phase to maximum knee extension, *TO* toe off

#### Biomechanical Determinants of Performance

Greater performance during the heavy sled pull was characterized by an increased stride length, stride rate and reduced ground contact time [32]. HP athletes also generally exhibited a more vertical trunk position and greater knee extension at foot strike [32]. Measures of flexed arm girth, mid-thigh girth, and total system force (calculated as the sum of the athlete’s body mass and 1RM squat) were reported to be the strongest anthropometric determinants of performance in the vehicle pull (flexed arm girth, *r* = 0.74; mid-thigh girth, *r* = 0.70; total system force, *r* = 0.68) [8].

#### Within Exercise Biomechanical Differences

The maximum velocity phase of the heavy sled pull was associated with greater stride length, knee extension at foot strike [32, 38], swing time, and a more horizontal trunk and vertical thigh position at foot strike and toe off [32] than the sub-maximal velocity and acceleration phase. Conversely, the initial stride had a greater mean resultant anterior/posterior and mean resultant medial/lateral ground reaction forces than strides at 2–3 m [38].

#### Between Exercise Biomechanical Differences

The back squat involved statistically greater hip and knee ROM than the heavy sled pull [38]. Greater knee flexion and a more vertical trunk position at the start of the concentric phase, and greater extension of the hip and knee at the point of maximum knee extension were also recorded during the squat [38]. The distinct differences in body positioning for the back squat vs. sled pull were supported by greater peak and mean vertical force during the back squat, and statistically greater peak and mean anterior force during the heavy sled pull [38].

### Static Lifting Exercises

In line with the terminology used in the sport of strongman, exercises where the athlete remains situated in the same general location throughout the exercise are typically categorized as static lifts. The static lifting strongman exercises biomechanically analyzed were the atlas stone lift, log lift, and tire flip. The log lift was the most studied static lifting exercise enabling within and between study comparison (Table 5).

**Table 5:** Static lift significant result comparisons

	Winwood et al. [39]		Renals et al. [37]			McGill et al. [35]		Keogh et al. [33], McGill et al. [35]
	165 mm diam log	Barbell clean and press	250 mm diam log	316 mm diam log	Barbell push press	Log lift	Atlas stone	Tire flip
Temporal								
Duration (s)	7.96 ± 3.77 (TD)	6.20 ± 1.96 (TD)	0.22 ± 0.02(PR)	0.22 ± 0.02(PR)	0.22 ± 0.03 (PR)	–	–	0.38 ± 0.17 ^☼^(SP)(HP)
			0.67 ± 0.06 (TD)	0.64 ± 0.07 (TD)	0.54 ± 0.47 (TD)			1.49 ± 0.92 (SP)(LP)
Kinematic								
Dip depth (cm)	17.4 ± 4.40 (PP)	18.0 ± 6.60 (PP)	14.0 ± 3.00* (PP)	13.0 ± 2.00* (PP)	17.0 ± 4.00 (PP)	–	–	–
Vertical lift velocity (m/s)	0.60 ± 0.10* (FP)	0.75 ± 0.15 (FP)	–	–	–	–	–	–
	1.06 ± 0.41* (SP)	1.69 ± 0.15 (SP)	–	–	–	–	–	–
	0.88 ± 0.07 (PP)	0.97 ± 0.08 (PP)	0.64 ± 0.07* (PP)	0.62 ± 0.06* (PP)	0.74 ± 0.07 (PP)	–	–	–
Hip angle (°)	52.0 ± 6.00* (LO)	60.0 ± 6.00 (LO)	–	–	–	–	–	–
	182 ± 5.00* (TR)	158 ± 15.0 (TR)	–	–	–	–	–	–
HIP ROM (°)	126 ± 9.00* (EL)	116 ± 10.0 (EL)	–	–	–	–	–	–
Knee angle (°)	99.0 ± 25.0* (SSP)	140 ± 11.0 (SSP)	–	–	–	–	–	–
	139 ± 11.0* (TR)	125 ± 13.0 (TR)	–	–	–	–	–	–
Trunk angle (°)	106 ± 2.00* (TR)	91.0 ± 6.00 (TR)	–	–	–	–	–	–
	93.0 ± 5.00* (BD)	87.0 ± 2.00 (BD)	–	–	–	–	–	–
Trunk ROM (°)	83.0 ± 8.00* (EL)	67.0 ± 12.0 (EL)	–	–	–	–	–	–
Kinetic								
Braking mean force (N)	–	–	680 ± 262 (PP)	625 ± 252* (PP)	775 ± 317 (PP)	–	–	–
Braking impulse (N.s)	–	–	116 ± 28.7* (PP)	106 ± 27.8* (PP)	131 ± 27.3 (PP)	–	–	–
Braking mean power (W)	–	–	− 943 ± 281* (PP)	− 854 ± 276* (PP)	− 1090 ± 283 (PP)	–	–	–
Mean posterior force (N)	− 67.0 ± 14.0* (EL)	− 91.0 ± 27.0 (EL)	–	–	–	–	–	–
Propulsive mean force (N)	–	–	3230 ± 357* (PP)	3130 ± 363* (PP)	3400 ± 492 (PP)	–	–	–
Propulsive impulse (N.s)	307 ± 56.8 (PP)	346 ± 66.8 (PP)	255 ± 38.8* (PP)	241 ± 28.7* (PP)	293 ± 40.0 (PP)	–	–	–
Propulsive mean power (W)	1920 ± 591* (PP)	2960 ± 802 (PP)	2040 ± 377* (PP)	1900 ± 295* (PP)	2470 ± 482 (PP)	–	–	–
Musc ant/post shear (N)	–	–	–	–	–	2800^§^	–	2600^§^
Musc comp load (N)	–	–	–	–	–	7500^§^	–	8800^§^
Musc ax twist stiff (Nm/rad)	–	–	–	–	–	25,300^§^	–	31,400^§^
Musc flex/ext stiff (Nm/rad)	–	–	–	–	–	32,400^§^	–	38,600^§^
Muscle activation (%MVC)								
Right rectus abdominis	–	–	–	–	–	27.3 ± 27.8^‡^	77.6 ± 41.6	87.8 ± 63.9^†^
Right external oblique	–	–	–	–	–	61.5 ± 49.1^‡^	97.6 ± 67.7	107 ± 45.4^†^

All data are reported as means ± standard deviation, unless specified otherwise. *Significant difference to barbell, ^†^significant difference to log lift, ^‡^significant difference to tire flip, ^☼^significant difference to lower performing athletes, ^§^value only provided in graph form and as such are approximate values with no standard deviation, *Ant/post* anterior/posterior, *ax* axial, *BD* bottom of dip, *comp* compressive, *diam* diameter, *EL* entire lift, *flex/ext* flexion/extension, *FP* first pull, *HP* higher performing athlete, *LO* lift off, *LP* lower performing athlete, *musc* muscle, *MVC* maximum voluntary contraction, *PP* push press phase, *PR* propulsive duration, *SP* second pull, *SSP* start of second pull, *stiff* stiffness, *TD* total lift duration, *TR* top retrieve phase

#### Biomechanical Determinants of Performance

The greatest biomechanical determinant of performance in the tire flip was observed as the second pull phase time (defined as the time between the tire passing the knee to the hands first leaving the tire), accounting for ~ 67% of the between-group (HP vs. LP) difference in total tire flip time [33]. Measures of calf girth, flexed arm girth and total system force (calculated as the sum of the athlete’s body mass and 1RM squat) were reported to be the strongest anthropometric determinants of performance in the tire flip (calf girth, *r* = 0.67; flexed arm girth, *r* = 0.66; total system force, *r* = 0.81) and log lift (calf girth, *r* = 0.75; flexed arm girth, *r* = 0.68; total system force, *r* = 0.71) [8].

#### Within Exercise Biomechanical Differences

No statistical analysis was performed comparing within exercise (between phase) biomechanical differences in any of the static type strongman lifts.

#### Between Exercise Biomechanical Differences

Winwood et al. [39] compared the biomechanics occurring during the clean and jerk/press movement when using a barbell and a log at a load of 70% of the athlete’s barbell clean and jerk 1RM. Renals et al. [37] compared the biomechanics occurring during the push press when using a barbell and logs of 250 mm and 316 mm diameter at a load of 65% of the athletes’ barbell push press 1RM. Greater knee flexion was observed during the start of the second pull phase (deep squat position with log resting on the thighs) of the log clean and press than the equivalent phase of the barbell clean and jerk. Greater knee and hip extension, and a more vertical trunk position occurred during the top retrieve phase (full standing position with log resting on top of chest) of the log clean and press than the equivalent phase of the barbell clean and jerk [39]. The increased flexion and extension lead to a greater trunk and hip ROM throughout the entire log clean and press movement than the barbell clean and jerk movement [39].

Statistically greater mean vertical velocities were reported during the first and second pull phases of the barbell clean and jerk when compared with the log clean and press, with no statistical differences in velocity or dip depth reported during the push press phase using either the barbell or log [39]. Renals et al. [37] did however report statistically greater vertical propulsive velocity and dip depth when using the barbell than the two different diameter logs during the push press. Braking and propulsive impulse, mean force and mean power were all reported to be statistically greater during the push press when using the barbell than the two logs [37]. Mean posterior force (backward, horizontally directed force) was observed to be statistically greater throughout the entire clean and jerk/press movement when using the barbell as opposed to the log [39].

The only reported differences in lumbar joint kinematics during static lifting exercises was greater lateral bend and twist during the tire flip (lateral bend: ~ 7°, twist: ~ 8°) than the log lift (lateral bend: ~ 3°, twist: ~ 6°) [35]. When spine angle was normalized to maximum spinal angle, only peak twist (tire flip: ~ 109%, log lift: ~ 68%) was reported to be statistically different [35]. Although no statistical differences in muscular and joint loading were reported between the static lifting exercises, anterior core muscle activation (right rectus abdominis, right external oblique) were reportedly greater in the tire flip than the log lift [35].

## Discussion

Existing literature provides a basic understanding of the biomechanics of a range of strongman exercises, with few studies extending to identify the biomechanical determinants of performance of strongman exercises [32–34]. Currently, there exists very little research evidence regarding how performance in one strongman event may be related to other strongman events. The only study that has empirically set out to answer this question has examined relationships between performance in strongman exercises, strength in TWTE and anthropometrics, with a limited number of strong correlations found between performance in individual strongman exercises (competition events) [8]. A limitation in the ability to answer this question is the wide variety of strongman event requirements, where various implements may be used for a given general movement pattern (e.g., for overhead pressing logs of varying diameters, axels, kegs, dumbbells, Viking machines can all be used), all of which can be performed to different competition requirements (as many repetitions as possible (AMRAP), 1RM, increasing load). As such, the following sections of the discussion will be organized under the general movement patterns of carrying/walking, pulling and static lifts. Qualitative analysis of strongman exercises in their most general form, along with quantitative results from studies of similar TWTE and CEA, may provide a greater understanding of strongman exercise performance determinants, injury risk and wider applications to other populations.

### Carrying/Walking Exercises

#### Bilateral Load Carriage

The farmer’s walk and yoke walk are the most common bilateral carrying strongman exercises used in strongman training [10, 18]. Little biomechanical analysis has been performed on the yoke walk, with spinal motion, muscle activation and loading being measured [35]. Although differing in the absolute load and positioning of the load being carried, quantitative analysis of the farmer’s walk and other forms of load carriage may provide a greater understanding of the biomechanics of the yoke walk strongman exercise.

A systematic review comparing the biomechanics of backpack load carriage and unloaded walking showed backpack load carriage to be associated with an increase in stride rate (ES = 0.37) and a decrease in stride length (ES = − 0.32) when compared with unloaded walking [40]. The effect of backpack load carriage on spatiotemporal measures across the studies was small; however, effect sizes progressively increased as load increased [40]. Such findings are consistent with the farmer’s walk exercise, where substantially greater loads were used and greater differences existed compared with unloaded walking (stride rate: ES = 4.20, stride length: ES = − 3.40) [7]. Although a physical limit will be approached, whereby the athlete is no longer able to increase their stride rate with a decrease in stride length, it may be expected that the greater loads that can be used in the yoke walk when compared with the farmer’s walk would result in further increases in stride rate and decreases in stride length.

Statistically greater anterior/posterior, medial/lateral and vertical ground reaction forces were reported during the farmer’s walk when compared with unloaded walking [7]. Similar results have been reported when comparing backpack load carriage with unloaded walking, where greater propulsive and braking (anterior/posterior), and vertical ground reaction forces were reported during backpack load carriage [40]. The difference in anterior/posterior ground reaction forces observed between unloaded walking and backpack load carriage may partially be the result of the center of mass of the carrier being pulled backward when the load is positioned posterior to the centerline of the body.

Similar to the farmer’s walk, the athlete’s ability to maintain/minimize the reduction in their stride length while maintaining or increasing their stride rate during the yoke walk will result in a higher velocity and thus a greater performance outcome by the athlete [34]. Where greater braking and propulsive forces have been reported in the farmer’s walk than unloaded walking conditions [7], it would be suggested that greater performance in the farmer’s walk and yoke would be achieved by minimizing any potential increases in braking force while maximizing increases in propulsive force. A limiting factor contributing to the athlete’s ability to demonstrate these biomechanical determinants of performance in the farmer’s walk may be the grip strength of the athlete, as in most competitions the only artificial aid athletes can use to assist their grip is lifting chalk. Similarly, a limiting factor contributing to the athlete’s ability to demonstrate these biomechanical characteristics in the yoke walk may be the athlete’s ability to brace their trunk and hip musculature and tolerate high compressive loads [35]. The use of load carriage exercises in strength and conditioning programs of non-strongman athletes may support the development of these limiting factors, whereby it has been reported that exercises such as the farmer’s walk are typically included in programs to develop grip strength and total body strength [10].

#### Unilateral Load Carriage

The keg walk technique adopted in McGill et al. [35], whereby the keg is carried on a single shoulder, is just one technique which may be used by an athlete in a keg walk competition event or as a strength training exercise for non-strongman athletes. Other techniques to perform the keg walk may include; wrapping one’s arms around the keg in a hugged position on the anterior surface of their abdomen, lifting and carrying the keg using the handles positioned around the rim of the keg, or a combination of the aforementioned techniques. Individualized biomechanical analysis of each technique would therefore be required and as such is beyond the scope of this review.

Performance in the suitcase carry has been characterized by an athlete’s ability to maintain a vertical spinal posture (with respect to the frontal and sagittal anatomical plane) and a constant step cadence [31]. This may be deduced by the tendency for an increase in lateral bend and inability to maintain a set cadence as load is progressively increased [31]. As the load used in previous suitcase (McGill et al. [35]: ~ 31% bodyweight, Holmstrup et al. [31]: ~ 63% bodyweight) and unilateral dumbbell carriage studies may be less than what is expected to be used in strongman training, trunk bend, lumbar spinal loading, ground reaction force asymmetry, and changes in gait characteristics may be further magnified in a true strongman setting where greater loads are carried.

Future research on the biomechanics of strongman bilateral and unilateral carrying/walking type exercises may assist in determining the biomechanical demands of military physical fitness assessment exercises. Such assessments include the jerry can carry which is used to assess grip strength and load carriage speed, whereby military personnel carry jerry cans (usually of mass > 20 kg) a short distance (~ 20 m) in the fastest possible time [41]. Research into the biomechanical demands of the yoke walk may provide a foundation for future research into the demands placed on firefighters carrying breathing apparatus and firefighting equipment, and trail porters who have been known to carry loads of one-and-a-half times their body mass over vast distances [42]. As the practical guidelines on how to best condition these occupational groups for load carriage are limited, findings from strongman research may play a pivotal role in this process [43].

### Pulling Exercises

Previous strongman biomechanical studies have only analyzed the biomechanics of athletes performing the heavy sled pull (> 100% body mass), which is typically used as a training tool to simulate the vehicle pull for strongman athletes, or as a strength and conditioning tool for other athletic groups [10, 32, 38]. Assessing the biomechanical similarities between persons performing the heavy and sub-body mass sled pull may assist in establishing the likely biomechanics of performing a vehicle pull.

Greater decreases in velocity, stride length and second-stride swing time, and greater increases in ground contact time have been found to occur when performing a sub-body mass sled pull at a sled load of 32.2% body mass compared with 12.6% body mass [44]. While no statistical difference in stride rate was reported between the two sub-body mass loading conditions, stride rate was statistically lower under both loading conditions than the unloaded condition [44]. No comparisons between loading conditions were made in the heavy sled pull study of Keogh et al. [32]; however similar changes in spatiotemporal parameters may be deduced from the lower velocity trials, whereby a reduced stride length and swing time, and increased ground contact time were reported [32].

When comparing joint kinematics between unloaded sprinting, sled pulls at 15%, 20%, 30%, and 40% body mass, statistically significant increases in knee and hip flexion at foot strike and toe off have been reported with an increase in sled load [45]. The greater knee and hip flexion at foot strike and toe off would likely result in the athlete attaining a more horizontal trunk position throughout the pull and increase the time and range of motion over which force was applied. Where the increases in sled mass in the study by Monte et al. [45] were associated with a decreased pull velocity, lower velocity during the heavy sled pull in Keogh et al. [32] and Winwood et al. [38] was similarly characterized by a more horizontal trunk position and greater knee flexion at foot strike. Qualitatively, it may appear that the more horizontal trunk orientation is a mechanism employed by the athlete to position the body so to optimize horizontal propulsive force production; however, more quantitative research is required to confirm this hypothesis.

The direction of the resultant ground reaction force of the athlete when performing the sled pull and strongman vehicle pull may also be dependent on the location at which the load is applied to the athlete’s body. A waist attachment site as opposed to a chest height attachment site on the athlete has been observed to result in the athlete attaining a more horizontal body position [46]. This is achieved through a greater trunk ROM and greater peak knee flexion during the stance phase of the sled pull [46]. A limiting factor when using a chest harness may be the increase in resistive trunk and hip extensor moment acting on the athlete. The vehicle pull strongman event is typically performed using a chest harness where the attachment site is located somewhere between the shoulder and the waist. To overcome the greater trunk and hip extensor moments and impart a greater horizontally directed propulsive force and impulse, the strength of the athlete’s trunk and hip flexors and their ability to maintain a predominantly horizontal position may both be determining factors of performance in the vehicle pull exercise.

From existing literature comparing the biomechanics of athletes performing sub-body mass sled pulls at varying loads, and the limited literature available on the heavy sled pull strongman exercise, it may be deduced that decreases in stride length and stride rate and increased trunk lean may be further magnified in the strongman vehicle pull where an increased resistive load is expected. Based on this knowledge and the relationship between increased sled load and decreased pulling velocity, it is suggested that greater performance in the strongman vehicle pull competition event may be characterized by the athlete’s ability to optimize the relationship between cadence and stride length, while attaining a total body position that enables greatest horizontal force production.

In addition to the heavy sled pull, strength and conditioning coaches often use a variety of similar resistive sprint training tools for the development of greater horizontal force production and sprinting ability in athletes [47, 48]. Such tools may include weighted vests, tires, and parachutes. Where such traditional forms of resistive towing training typically rely on ground reaction forces initially generated through the lower body, a strongman vehicle pull typically also includes the use of a thick rope in which the upper body musculature assists in the pull. The training benefits of simultaneous lower and upper body force application, as seen in the vehicle pull, may be particularly relevant in such sports as rowing and kayaking where simultaneous lower body pushing and upper body pulling forces are required. Another benefit may be seen during the competitive phases of an annual periodized plan, whereby athletes have reduced time to devote to strength and conditioning. At such times, total body exercises such as the truck pull and push press may allow high kinetic outputs to be generated through the primary upper and lower body musculature within the one exercise [49].

The current heavy sled pull research may be used as a basis for further research into the biomechanical demands of performing other variations of pulling type exercises such as the backward drag. The backward drag technique is used in firefighting and military physical fitness assessments and service, where service people may be required to drag victims out of danger [50, 51]. Further investigation into the biomechanical demands of a vehicle pull may be of benefit to military operations, as soldiers may be faced with instances where they are required to pull/push heavy equipment over short distances [51].

### Static Lifting Exercises

A relative lack of quantitative biomechanical analysis exists on static lifts such as the atlas stone lift, log lift, and tire flip. To qualitatively analyze these three exercises, they may be broken down into phases and biomechanically analyzed alongside a variety of different TWTE and CEA.

#### Atlas Stone Lift

Of the three static strongman lifts analyzed in the current literature, the atlas stone may be seen as one of the most mechanically demanding and potentially injurious strongman exercises [4]. Quantitatively, little biomechanical analysis has been performed on the atlas stone lift, with just joint/muscle loading and muscle activation being measured in one study of three athletes [35].

In phase one of the atlas stone lift, the athlete attempts to lift the stone off the ground using a “hugged” grip and a lifting technique similar to a Romanian deadlift. Once the stone is off the ground, the athlete assumes a paused position with the stone resting in the lap. The most similar and comprehensive field of research related to the biomechanics of this movement is in the area of injury risk assessment/prevention for the manual handling stoop lifting technique, which is characterized by a bent back and straight knee posture until lift completion (fully erect standing position) [52]. Net moments and compressive forces acting on the spine have been reported to be similar between the stoop lifting technique and the often preferred squat lifting technique (characterized by a straight back and bent knee posture until lift completion) [52]. The insignificant differences in joint loading between these lifting techniques are supported by the findings of McGill et al. [35] where vertebral joint moments, muscular/joint compression and shear forces during the atlas stone lift were reported to be of a similar or lower magnitude to other strongman exercises analyzed including the farmer’s walk, yoke walk, keg walk and log lift.

The explosive movement initiated from the paused position at the end of phase one to the quarter-squat position at the end of phase two of the atlas stone lift may be the most similar to that of the beginning of the concentric phase of the box squat [53]. The box squat has been reported to result in statistically lower peak force production than the powerlifting or traditional style squat (box squat: 2528 ± 302, powerlifting: 2685 ± 301, traditional squat: 2680 ± 309 N) [53]. This was suggested to be due to the pause and transfer of load from the system to the box at the bottom of the squat, meaning the box squat may lose some of the benefits of the stretch-shorten cycle in terms of force production and loads lifted. There was however a statistically greater rate of force development in the box squat compared with the traditional and powerlifting style squats [53].

In phase three of the atlas stone lift, the athlete moves with the stone from a quarter-squat position to a full extension standing position, with the stone being transferred to a chest height ledge or over a bar. This final stage of the atlas stone lift may show biomechanical similarity to the concentric phase of the front squat, whereby the load is lifted in a squat like position on the anterior surface of the body to a full extension standing position. While still being in a flexed torso position throughout the entirety of the front squat, during the final stage of the atlas stone lift, the athlete often moves into a position of torso extension when the stone is passed onto the ledge/over the bar. The degree of movement of the torso into an extended state may be expected to differ between athletes of varying anthropometrics and performance standard, and may result in unique muscular and joint loading. Further quantitative analysis of the atlas stone lift is required to confirm this hypothesis.

It may be suggested that increasing a strongman athlete’s ability to utilize the stretch-shorten cycle in the transition from the end of phase one (bottom of squat) to the initial stages of phase two, while also promoting a high rate of force development throughout phase two, may be key in achieving greater performance in the atlas stone lift. Although a tacky substance is often used by athletes to assist in gripping the stone, a likely limiting factor of performance in the atlas stone lift may be the hugging grip strength of the athlete to initialize the lift of the stone off the ground during phase one.

The inclusion of the atlas stone lift into a strength and conditioning program may be of interest to military personnel or civilians required to perform physical lifting fitness assessments. Such an example of this form of test is the box lift and place assessment conducted in the Australian Army, whereby a box (up to 40 kg) must be lifted from the ground and placed on a ledge of height 1.5 m [54]. Where atlas stones may not be available at many strength and conditioning facilities, sandbags and heavy medicine (slam) balls may be lifted using a similar technique to the atlas stone lift.

#### Log Lift

The log lift is commonly used by strength and conditioning coaches as an alternative to traditional overhead lift variations, with the biomechanics of persons performing the log lift being relatively well analyzed compared with other strongman events [10, 35, 37, 39]. Existing literature has demonstrated not only the similarities of the log lift to the clean and jerk and push press but also the profound mechanical differences, where greater joint ROM was reported in the log variations, and greater force and impulses were reported in the barbell variation [37, 39]. These differences may be attributed to the log size and shape, as well as the training background of the athletes in these studies. There however remains a gap in the current strongman literature identifying the biomechanical determinants of greater performance in the log lift exercise. To the authors knowledge, literature on the biomechanical determinants of performance in all forms of strength-based overhead pressing exercises is lacking. As such, the advancement of researchers’ understandings of the biomechanical determinants of performance in the log lift is limited to phase one (movement of the log from the ground to a knee height or lap position) and phase two (movement of the log from a knee height/lap position to a racked position of the chest), which may exhibit some similarities to the power clean [39].

A greater 1RM in the power clean has been reported to be achieved by athletes that could minimize hip ROM during the first pull phase of the lift and produce a greater rapid extension of the hip during the second pull phase (*r* = 0.87) [55]. An athlete’s ability to execute the clean with minimal hip flexion during phase one and perform a powerful hip extension during phase two of the log lift is particularly evident in log lift competition events that require athletes to perform AMRAP in an allocated time at sub-maximal loads. These first two phases of the log clean appear kinematically similar to that of the atlas stone lift, suggesting there might be some commonalities in the determinants of performance of these two strongman lifts.

Greater performance (achievement of a successful lift at a greater barbell load) in the power clean has also been characterized by an athlete’s ability to keep the barbell close to their body throughout the second pull phase of the lift (likely through a greater net force application toward the body) [56]. This is likely to have great applicability to the log lift as the increased diameter of the log is expected to make achieving greater net force application toward the body more difficult due to the center of mass of the log being positioned further in front of the center of mass of the athlete.

Strength and conditioning coaches may consider lighter variations of the log lift exercise where the training of overhead vertical strength is required in an injury rehabilitation program or where an athlete has a history of shoulder instability. It has been suggested that using a neutral grip with the hands positioned shoulder width apart, as used in the log lift, promotes an anatomically optimal position for overhead pressing [57]. It must however be acknowledged that the greater diameter of the log compared with the bar may increase the lumbar loads during the log lift compared with barbell overhead lifts. As a result, the strength and conditioning coach may need to take into account an individual athlete’s injury history, movement competency, physical capacities, and sporting demands before determining the risks and rewards of these exercises.

#### Tire Flip

Although the tire flip is commonly used as a strength and conditioning training tool at a recreational and elite sporting level for power, strength, endurance, and metabolic conditioning training [10], biomechanical analysis of the exercise has only considered temporal determinants of greater performance [33]. Biomechanical analysis of the tire flip exercise may be particularly difficult as the lift is one of the few strongman lifts where the implement remains in contact with the ground throughout the entirety of the lift, thus quantifying the load lifted by the athlete may be difficult. Additionally, the dimension, mass and frictional characteristics of the tire and ground are likely to have an impact on the technique/biomechanics of the athlete performing the lift.

In phase one of the tire flip, the athlete begins the movement by lifting one side of the tire off the ground to just above the knee. This would appear to be biomechanically similar to aspects of the initial lifting phase of the conventional and sumo deadlift. The greater horizontally oriented trunk position at lift off during the tire flip may be more biomechanically similar to the trunk position at lift off during the conventional deadlift, when compared with the sumo deadlift [58]. Conversely, the wide stance used at lift off during the tire flip may be more biomechanically similar to the stance width at lift off during the sumo deadlift, when compared with the conventional deadlift [58].

In the second pull phase of the tire flip, the athlete moves the tire from the knee height position to the point of first hand release where the tire may be close to a 75° angle from horizontal. This phase of the tire flip may be biomechanically similar to the power clean, whereby the phase is characterized by a rapid acceleration of the implement from just above the knee, that primarily results from forceful triple extension of the ankle, knee and hip joints [33, 56]. Comparing successful versus unsuccessful attempts of the power clean revealed that greater performance may be characterized by an athlete’s ability to minimize forward barbell displacement during the second pull phase of the lift [56]. It is expected that the reduced forward barbell displacement relative to the centerline of the body minimizes the resistive joint torques experienced by the athlete and thus ensures maximal vertical force production.

In the third phase of the tire flip, the athlete catches the tire at approximately chest height by attaining a position of full extension of the wrist and hand, pronation of the forearm, flexion of the elbow and extension of the shoulder, before powerfully pushing the tire past its tipping point. The closest biomechanical assessment of such pushing movement may be that of a loaded cart (181 kg), where a decrease in horizontal force production as the height of force application increased (from a standing knuckle, elbow or shoulder height) was reported [59]. The tipping motion of the tire flip is however expected to result in profound biomechanical differences when compared with the translational/rolling motion of a cart. While the first two phases of the tire flip are expected to require a substantially vertical force component, it is expected that as the flip progresses to the third phase, the requirement of a greater horizontal force component becomes apparent.

The second pull phase time of the tire flip has already been identified as a key determinant of performance [33]. The athlete’s ability to move their body toward the tire and maintain or further advance (minimize) their body position relative to the tire during phase two is expected to be key factor in achieving a shorter second pull phase time. The ability to maintain or further advance one’s body position relative to a resistive load may be of particular interest to strength and conditioning coaches of rugby and American football athletes, where athletes are often required to block an opposing player’s movement or advance on an opponent’s ground. Strength and conditioning coaches may consider the use of the tire flip as an effective tool for the training of greater horizontal force and power production for rugby and American football athletes.

## Limitations

One limitation of this systematic review may be the extent to which TWTE and CEA in the discussion section were identified. A systematic review style search and screening process could have been used to identify all relevant articles to exercises such as the clean and jerk, deadlift and squat. The results of such a process may have provided slightly greater insight into and discussion around the biomechanics of strongman exercises compared with these other resisted human movements. The extensive search and screening process was not undertaken as the authors deemed the likelihood of finding additional relevant literature to be relatively small. The second limitation of this systematic review is in the tool used for the risk of bias/quality assessment, whereby it may be seen that using a risk of bias/quality assessment tool developed by the author team may add an additional level of bias to the systematic review itself. The use of Google Scholar for forward citation tracking may be considered a minor limitation to the systematic review due to the unreliability sometimes associated with the platform.

## Conclusion

The collation, assessment, and interpretation of the results from the eleven identified strongman biomechanics studies have outlined the current understanding of the biomechanical determinants and applications of strongman exercises. Qualitative assessment of the eight strongman exercises and comparison with quantitative biomechanical data of TWTE and CEA were used to develop further insights into the determinants of strongman exercise performance and applications of strongman exercises outside of the sport of strongman. A lack of quantitative biomechanical data was identified in the areas of a basic biomechanical analysis of the yoke walk, unilateral load carriage exercises, vehicle pull, atlas stone lift, and tire flip and more specific biomechanical performance determinants of the log lift exercise. Future research in the identified areas of strongman biomechanics is expected to provide a greater understanding of the biomechanical determinants of performance in a wider range of strongman exercises, and the potential training adaptations and risks expected when performing and/or incorporating strongman exercises into strength and conditioning or injury rehabilitation programs. This review has demonstrated the likely applicability and benefit of current and future strongman exercise biomechanics research to strongman athletes and coaches; strength and conditioning coaches considering using strongman exercises in a training program; and tactical operators (e.g., military, army) and other manual labor occupations.

## Supplementary information

**Additional file 1: Table S1**. Search term strategy used for each database. (DOCX 22 kb)

## Data Availability

The search term strategy used for each database is provided in Supplemental Content 1.docx. Data sharing is not applicable to this publication as no datasets were produced or analyzed during the study.

## Abbreviations

1RM: One-repetition maximum
AMRAP: As many repetitions as possible
CEA: Common everyday activities
HAB: Hip abductor
HP: Higher performing
LP: Lower performing
MVC: Maximum voluntary contraction
PRISMA: Preferred Reporting Items for Systematic Reviews and Meta-Analyses
PROSPERO: The International Prospective Register of Systematic Reviews
ROM: Range of motion
TWTE: Traditional weight training exercises
