# Correction to: The biomechanics and applications of strongman exercises: a systematic review

**DOI:** 10.1186/s40798-020-0239-3

**Published:** 2020-02-05

**Authors:** Benjamin R. Hindle, Anna Lorimer, Paul Winwood, Justin W. L. Keogh

**Affiliations:** 1 Faculty of Health Sciences and Medicine, Bond University, Gold Coast, Australia; 2 Department of Sport and Fitness, Faculty of Community Wellbeing and Development, Toi Ohomai Institute of Technology, Tauranga, New Zealand; 3 Sports Performance Research Institute New Zealand (SPRINZ), AUT Millennium Institute, AUT University, Auckland, New Zealand; 4 Kasturba Medical College, Manipal Academy of Higher Education, Mangalore, Karnataka India; 5 Cluster for Health Improvement, Faculty of Science, Health, Education and Engineering, University of the Sunshine Coast, Sunshine Coast, Australia

**Correction to: Sports Med Open**

**https://doi.org/10.1186/s40798-019-0222-z**

The original article [1] contained an error whereby the symbol ‘☼’ originally included in and below Tables 2, 3, 4 and 5 did not display properly. This has been rectified in the following versions of these tables.

**Table 2:** Walking/carrying results comparisons - farmers walk

	Winwood et al. [7]			Keogh et al. [34]			
	Farmers walk	Unloaded walk	Effect size	Higher performer	Lower performer	Effect size	Group ave.
Spatiotemporal							
Ground contact time (s)	0.46 ± 0.06* (MVP)	0.67 ± 0.06 (MVP)	−3.50	0.29 ± 0.02† (MVP˟)	0.34 ± 0.03 (SVP˟)	−1.96	0.30 ± 0.03☼ (MVP)
	0.53 ± 0.09*(AP)	0.77 ± 0.07 (AP)	−3.00	0.39 ± 0.04† (AP)	0.32 ± 0.03 (AP)	1.98	0.36 ± 0.04 (AP)
Stride rate (Hz)	1.42 ± 0.17* (MVP)	0.88 ± 0.06 (MVP)	4.20	2.01 ± 0.13† (MVP˟)	1.83 ± 0.04 (SVP˟)	1.88	1.97 ± 0.13☼ (MVP)
	1.21 ± 0.12* (AP)	0.82 ± 0.04 (AP)	4.40	1.88 ± 0.10† (AP)	1.64 ± 0.12 (AP)	2.17	1.79 ± 0.14 (AP)
Stride length (m)	1.04 ± 0.12* (MVP)	1.43 ± 0.11 (MVP)	−3.40	1.83 ± 0.04† (MVP˟)	1.40 ± 0.17 (SVP˟)	3.48	1.67 ± 0.10☼ (MVP)
	0.85 ± 0.19* (AP)	1.33 ± 0.11 (AP)	−3.10	1.38 ± 0.16 (AP)	1.33 ± 0.09 (AP)	0.39	1.32 ± 0.12 (AP)
Average velocity (m/s)	1.48 ± 0.19 (MVP)	1.26 ± 0.15 (MVP)	1.28	3.66 ± 0.17† (MVP)	2.83 ± 0.36 (MVP)	2.95	3.29 ± 0.38☼ (MVP)
	1.05 ± 0.21 (AP)	1.11 ± 0.09 (AP)	−0.37	2.61 ± 0.38 (AP)	2.19 ± 0.27 (AP)	1.27	2.41 ± 0.32 (AP)
Kinematic							
Ankle angle at FS (°)	95.0 ± 3.00* (MVP)	105 ± 2.00 (MVP)	−3.80	101 ± 6.00† (SVP˟)	113 ± 5.00 (MVP˟)	−2.17	110 ± 9.00 (MVP)
	96.00 ± 6.00* (AP)	105 ± 2.00 (AP)	− 2.30	99.0 ± 8.00 (AP)	106 ± 6.00 (AP)	−0.99	100 ± 8.00 (AP)
Ankle angle at TO (°)	100 ± 5.00* (MVP)	115 ± 9.00 (MVP)	−2.10	118 ± 5.00 (MVP˟)	117 ± 7.00 (SVP˟)	0.16	114 ± 6.00(MVP)
	105 ± 6.00 (AP)	118 ± 5.00 (AP)	−2.30	108 ± 4.00† (AP)	114 ± 3.00 (AP)	− 1.70	111 ± 5.00 (AP)
Ankle ROM (°)	4.00 ± 4.00 (MVP)	10.0 ± 10.0 (MVP)	−0.70	−10.0 ± 4.00† (SVP)	1.00 ± 5.00 (MVP)	−2.43	−4.00 ± 7.00☼ (MVP)
Knee angle at FS (°)	154 ± 7.00* (MVP)	178 ± 6.00 (MVP)	−3.70	156 ± 6.00 (MVP˟)	166 ± 16.0 (SVP˟)	−0.83	155 ± 6.00☼ (MVP)
	150 ± 9.00* (AP)	174 ± 10.0 (AP)	−2.50	147 ± 7.00 (AP)	151 ± 5.00 (AP)	−0.66	150 ± 6.00 (AP)
Thigh angle at FS (°)	34.0 ± 6.00* (MVP)	23.0 ± 7.00 (MVP)	1.80	38.0 ± 3.00† (MVP˟)	31.0 ± 4.00 (SVP˟)	1.98	34.0 ± 3.00 (MVP)
Thigh ROM (°)	−19.0 ± 5.00 (MVP)	−22.0 ± 10.0 (MVP)	0.40	− 44.0 ± 4.00† (MVP˟)	− 35.0 ± 6.00 (SVP˟)	−1.77	− 38.0 ± 4.00☼ (MVP)
Trunk angle at FS (°)	78.0 ± 3.00* (MVP)	90.0 ± 2.00 (MVP)	− 4.10	–	–		–
	69.0 ± 5.00* (AP)	85.0 ± 2.00 (AP)	− 4.30	–	–		–
Trunk angle at TO (°)	76.0 ± 4.00* (MVP)	87.0 ± 2.00 (MVP)	− 3.20	–	–		–
	70.0 ± 5.00* (AP)	84.0 ± 4.00 (AP)	− 3.40	–	–		–
Kinetic							
Mean anterior GRF (N)	127 ± 31.0* (MVP)	83.0 ± 25.0 (MVP)	1.60	–	–		–
Peak anterior GRF (N)	447 ± 98.0* (MVP)	259 ± 53.0 (MVP)	2.40	–	–		–
Mean medial GRF (N)	120 ± 41.0* (MVP)	70.0 ± 36.0 (MVP)	1.30	–	–		–
Peak medial GRF (N)	241 ± 73.0* (MVP)	120 ± 62.0 (MVP)	1.80	–	–		–
Mean posterior GRF (N)	159 ± 45.0* (MVP)	94.0 ± 34.0 (MVP)	1.60	–	–		–
Peak posterior GRF (N)	389 ± 143* (MVP)	211 ± 77.0 (MVP)	1.50	–	–		–
Mean vertical GRF (N)	2540 ± 376* (MVP)	1030 ± 247 (MVP)	4.70	–	–		–
Peak vertical GRF (N)	3630 ± 608* (MVP)	1510 ± 387 (MVP)	4.10	–	–		–
Peak lateral GRF (N)	210 ± 73.0* (MVP)	119 ± 45.0 (MVP)	1.50	–	–		–

All data are reported as means ± standard deviation, unless specified otherwise. Effect sizes reported for between exercise [7] and between performance standard [34], A positive effect size indicates the left-hand column (farmers walk or higher performer) had a greater value than the respective right-hand column (unloaded walk or lower performer).* significant difference to unloaded walking, † significant difference to low performing athletes, ☼ significant difference to acceleration phase, ˟ comparison between phase based on distance, *AP* acceleration phase, *ave* average, *GRF* ground reaction force, *MVP* maximum velocity phase, *SVP* submaximal velocity phase

**Table 3:** Significant differences in muscle activation and kinetic outcomes between the walking/carrying exercises

	Farmers walk [35]	LH suitcase carry [35]	RH suitcase carry [35]	Yoke walk [35]
Muscle activity (%MVC)				
Left upper erector spinae	77.6 ± 29.3 ‡	47.1 ± 6.20	32.4 ± 4.60 *☼	69.3 ± 17.5 ‡
Right upper erector spinae	91.4 ± 54.7 †	24.9 ± 17.6 *‡☼	52.1 ± 17.3 †	65.6 ± 14.4†
Left lower erector spinae	106 ± 51.1 †	31.6 ± 10.1 *‡☼	77.4 ± 21.3 †	79.2 ± 10.2 †
Right lower erector spinae	144 ± 36.7 ‡	96.9 ± 20.4 ‡	44.1 ± 9.10 *†☼	107 ± 31.5 ‡
Left latissimus dorsi	169 ± 55.4 *‡	97.4 ± 55.7	68.9 ± 23.2 ☼	51.9 ± 26.4 ☼
Right latissimus dorsi	152 ± 26.7 *†	65.3 ± 6.20 ☼	91.4 ± 39.1	45.5 ± 31.7 ☼
Left external oblique	39.3 ± 30.6 †	12.6 ± 5.30 *‡☼	61.5 ± 21.9 †	47.5 ± 31.7 †
Right external oblique	50.4 ± 17.4 ‡	65.1 ± 24.4 ‡	29.0 ± 17.8 *†☼	58.8 ± 17.4 ‡
Right rectus abdominis	13.3 ± 3.80	14.6 ± 4.50	5.60 ± 1.80 *	22.3 ± 18.1 ‡
Right gluteus maximus	114 ± 70.3 ‡	78.2 ± 39.5	50.5 ± 31.2 *☼	113 ± 52.1 ‡
Right gluteus medius	108 ± 66.9 ‡	64.1 ± 38.7	57.3 ± 23.6 *☼	108 ± 69.7 ‡
Right bicep femoris	54.0 ± 13.7 †	31.2 ± 7.50 *‡ ☼	48.3 ± 8.6 †	61.7 ± 6.30 †
Right rectus femoris	77.4 ± 35.6	41.1 ± 9.20 *	56.5 ± 11.5 *	107 ± 23.5 †‡
Kinetic				
Muscular anterior/posterior shear (N)	2800 †	1680 ☼	1160	1890
Muscular compressive load (N)	7900 †	5800 *☼	6700	7800 †
Muscular axial twist stiffness (Nm/rad)	27,200 †‡	19,100 ☼	24,600 ☼	25,900
Muscular flexion/extension stiffness (Nm/rad)	35,600	24,000 *	27,500	38,600 †

All data are reported as means ± standard deviation, unless specified otherwise. * significant difference to yoke walk, † significant difference to left hand suitcase carry, ‡ significant difference to right hand suitcase carry, ☼ significant difference to farmers walk, *LH* left hand, *MVC* maximum voluntary contraction *RH* right hand

**Table 4:** Pulling significant results comparisons - heavy sled pull

	Winwood et al. [38]			Keogh et al. [32]			
	Heavy sled pull	Effect size	Back squat	Higher performer	Lower performer	Effect size	Group ave.
Spatiotemporal							
Ground contact time (s)	0.35 ± 0.04 (MVP)	−0.85	–	0.33 ± 0.04† (MVP)	0.76 ± 0.37 (MVP)	−1.63	0.48 ± 0.23 (MVP)
	0.38 ± 0.03 (AP)		–	0.42 ± 0.19 (AP)	0.57 ± 0.23 (AP)	−0.71	0.53 ± 0.32 (AP)
Stride rate (s)	1.42 ± 0.14 (MVP)	0.07	–	1.63 ± 0.12† (MVP)	1.10 ± 0.42 (MVP)	1.72	1.37 ± 0.39 (MVP)
	1.41 ± 0.14 (AP)		–	1.50 ± 0.55 (AP)	1.29 ± 0.37 (AP)	0.45	1.45 ± 0.50 (AP)
Swing time (s)	0.33 ± 0.04 (MVP)	0.39	–	0.29 ± 0.03 (MVP)	0.27 ± 0.05 (MVP)	0.49	0.28 ± 0.04☼ (MVP)
	0.31 ± 0.06 (AP)		–	0.28 ± 0.07† (AP)	0.23 ± 0.05 (AP)	0.82	0.25 ± 0.06 (AP)
Stride length (m)	1.29 ± 0.17☼ (MVP)	1.81	–	1.29 ± 0.26† (MVP)	0.80 ± 0.16 (MVP)	2.27	1.03 ± 0.26☼ (MVP)
	1.00 ± 0.15 (AP)		–	0.85 ± 0.25† (AP)	0.65 ± 0.04 (AP)	1.12	0.74 ± 0.28 (AP)
Kinematic							
Average velocity (m/s)	1.83 ± 0.22☼ (MVP)	2.44	–	2.08 ± 0.08† (MVP)	0.99 ± 0.50 (MVP)	3.04	1.61 ± 0.55☼ (MVP)
	1.39 ± 0.13 (AP)		–	1.22 ± 0.20† (AP)	0.79 ± 0.32 (AP)	1.61	1.04 ± 0.30 (AP)
Knee angle at FS (°)	114 ± 6.00☼ (MVP)	1.38	–	132 ± 9.00† (MVP)	112 ± 22.0 (MVP)	1.19	124 ± 18.0☼ (MVP)
	103 ± 9.00(AP)		–	125 ± 12.0† (AP)	110 ± 10.0(AP)	1.36	116 ± 13.0 (AP)
Knee angle at TO (°)	138 ± 14.0 (MVP)	0.35	–	153 ± 7.00 (MVP)	148 ± 10.0(MVP)	0.59	149 ± 9.00☼ (MVP)
	133 ± 14.0 (AP)		–	148 ± 14.0† (AP)	138 ± 17.0 (AP)	0.65	141 ± 15.0 (AP)
Thigh angle at FS (°)	–	–	–	23.0 ± 5.00† (MVP)	19.0 ± 5.0 (MVP)	0.80	21.0 ± 5.00☼ (MVP)
	–		–	14.0 ± 10.0 (AP)	16.0 ± 8.00 (AP)	−0.22	15.0 ± 10.0 (AP)
Trunk angle at FS (°)	61.0 ± 13.0 (MVP)	− 0.67	–	41.0 ± 7.00† (MVP)	8.00 ± 29.0 (MVP)	1.56	26.0 ± 24.0☼ (MVP)
	77.0 ± 30.0 (AP)		–	29.0 ± 17.0† (AP)	2.00 ± 16.0 (AP)	1.64	14.0 ± 21.0 (AP)
Trunk angle at TO (°)	61.0 ± 11.0 (MVP)	−0.49	–	41.0 ± 9.00† (MVP)	14.0 ± 25.0 (MVP)	1.59	28.0 ± 21.0☼ (MVP)
	69.0 ± 20.0 (AP)		–	31.0 ± 15.0† (AP)	10.0 ± 14.0 (AP)	1.45	19.0 ± 19.0 (AP)
Kinetic							
Mean anterior GRF (N)	555 ± 107* (SC to MKE)	6.63	43.0 ± 22.0 (SC to MKE)	–	–		–
Peak anterior GRF (N)	810 ± 174* (SC to MKE)	5.13	126 ± 73.0 (SC to MKE)	–	–		–
Mean vertical GRF (N)	1330 ± 364* (SC to MKE)	−2.38	2580 ± 648 (SC to MKE)	–	–		–
Peak vertical GRF (N)	1740 ± 463* (SC to MKE)	−1.85	3500 ± 1270 (SC to MKE)	–	–		–
Mean resultant ant/post force (N)	271 ± 89.0☼ (MVP)	−1.95	–	–	–	–	–
	526 ± 162 (AP)		–	–	–	–	–
Mean resultant med/lat force (N)	−5.00 ± 22.0☼ (MVP)	−1.75	–	–	–	–	–
	24.0 ± 8.00 (AP)		–	–	–	–	–

All data are reported as means ± standard deviation, unless specified otherwise. Spatiotemporal and kinematic effect sizes reported for between phase [38] and between performance standard [32], Kinetic effect sizes reported for between exercise (heavy sled pull vs back squat). A positive effect size indicates the left-hand column (higher performer or heavy sled pull) or top row (maximum velocity phase) had a greater value than the respective right-hand column (lower performer or back squat) or bottom row (acceleration phase).* significant difference to back squat, † significant difference to low performing athletes, ☼ significant difference to acceleration phase, *ant/post* anterior/posterior, *AP* acceleration phase, *ave* average, *FS* foot strike, *GRF* ground reaction force, *med/lat* medial/lateral, *MVP* maximum velocity phase, *SC to MKE* start of concentric phase to maximum knee extension, *TO* toe off

**Table 5:** Static lift significant result comparisons

	Winwood et al. [39]		Renals et al. [37]			McGill et al. [35]		Keogh et al. [33], McGill et al. [35]
	165 mm diam log	Barbell clean & press	250 mm diam log	316 mm diam log	Barbell push press	Log lift	Atlas stone	Tire flip
Temporal								
Duration (s)	7.96 ± 3.77 (TD)	6.20 ± 1.96 (TD)	0.22 ± 0.02(PR)	0.22 ± 0.02(PR)	0.22 ± 0.03 (PR)	–	–	0.38 ± 0.17 (SP)(HP) ☼
			0.67 ± 0.06 (TD)	0.64 ± 0.07 (TD)	0.54 ± 0.47 (TD)			1.49 ± 0.92 (SP)(LP)
Kinematic								
Dip depth (cm)	17.4 ± 4.40 (PP)	18.0 ± 6.60 (PP)	14.0 ± 3.00* (PP)	13.0 ± 2.00* (PP)	17.0 ± 4.00 (PP)	–	–	–
Vertical lift velocity (m/s)	0.60 ± 0.10* (FP)	0.75 ± 0.15 (FP)	–	–	–	–	–	–
	1.06 ± 0.41* (SP)	1.69 ± 0.15 (SP)	–	–	–	–	–	–
	0.88 ± 0.07 (PP)	0.97 ± 0.08 (PP)	0.64 ± 0.07* (PP)	0.62 ± 0.06* (PP)	0.74 ± 0.07 (PP)	–	–	–
Hip angle (°)	52.0 ± 6.00* (LO)	60.0 ± 6.00 (LO)	–	–	–	–	–	–
	182 ± 5.00* (TR)	158 ± 15.0 (TR)	–	–	–	–	–	–
HIP ROM (°)	126 ± 9.00* (EL)	116 ± 10.0 (EL)	–	–	–	–	–	–
Knee angle (°)	99.0 ± 25.0* (SSP)	140 ± 11.0 (SSP)	–	–	–	–	–	–
	139 ± 11.0* (TR)	125 ± 13.0 (TR)	–	–	–	–	–	–
Trunk angle (°)	106 ± 2.00* (TR)	91.0 ± 6.00 (TR)	–	–	–	–	–	–
	93.0 ± 5.00* (BD)	87.0 ± 2.00 (BD)	–	–	–	–	–	–
Trunk ROM (°)	83.0 ± 8.00* (EL)	67.0 ± 12.0 (EL)	–	–	–	–	–	–
Kinetic								
Braking mean force (N)	–	–	680 ± 262 (PP)	625 ± 252* (PP)	775 ± 317 (PP)	–	–	–
Braking impulse (N.s)	–	–	116 ± 28.7* (PP)	106 ± 27.8* (PP)	131 ± 27.3 (PP)	–	–	–
Braking mean power (W)	–	–	− 943 ± 281* (PP)	− 854 ± 276* (PP)	− 1090 ± 283 (PP)	–	–	–
Mean posterior force (N)	−67.0 ± 14.0* (EL)	− 91.0 ± 27.0 (EL)	–	–	–	–	–	–
Propulsive mean force (N)	–	–	3230 ± 357* (PP)	3130 ± 363* (PP)	3400 ± 492 (PP)	–	–	–
Propulsive impulse (N.s)	307 ± 56.8 (PP)	346 ± 66.8 (PP)	255 ± 38.8* (PP)	241 ± 28.7* (PP)	293 ± 40.0 (PP)	–	–	–
Propulsive mean power (W)	1920 ± 591* (PP)	2960 ± 802 (PP)	2040 ± 377* (PP)	1900 ± 295* (PP)	2470 ± 482 (PP)	–	–	–
Musc ant/post shear (N)	–	–	–	–	–	2800§	–	2600§
Musc comp load (N)	–	–	–	–	–	7500§	–	8800§
Musc ax twist stiff (Nm/rad)	–	–	–	–	–	25,300§	–	31,400§
Musc flex/ext. stiff (Nm/rad)	–	–	–	–	–	32,400§	–	38,600§
Muscle activation (%MVC)								
Right rectus abdominis	–	–	–	–	–	27.3 ± 27.8‡	77.6 ± 41.6	87.8 ± 63.9†
Right external oblique	–	–	–	–	–	61.5 ± 49.1‡	97.6 ± 67.7	107 ± 45.4†

All data are reported as means ± standard deviation, unless specified otherwise. * significant difference to barbell, † significant difference to log lift, ‡ significant difference to tire flip, ☼ significant difference to lower performing athletes, § value only provided in graph form and as such are approximate values with no standard deviation, *Ant/post* anterior/posterior, *ax* axial, *BD* bottom of dip, *comp* compressive, *diam* diameter, *EL* entire lift, *flex/ext* flexion/extension, *FP* first pull, *HP* higher performing athlete, *LO* lift off, *LP* lower performing athlete, *musc* muscle, *MVC* maximum voluntary contraction, *PP* push press phase, *PR* propulsive duration, *SP* second pull, *SSP* start of second pull, *stiff* stiffness, *TD* total lift duration, *TR* top retrieve phase
