# Supplementary material for: The Biomechanics and Applications of Strongman Exercises: a Systematic Review
Source: Sports Med Open. 2019 Dec 9;5:49. doi: 10.1186/s40798-019-0222-z (PMC6901656; doi:10.1186/s40798-019-0222-z)
Supplement: Supplementary file 1 — Additional file 1: Table S1. Search term strategy used for each database. (DOCX 22 kb) [file 40798_2019_222_MOESM1_ESM.docx]

Additional file 1: Table S1: Search term strategy used for each database

| **Ausport Med** | (strongman OR "strong man" OR "strong-man" OR junkyard OR "junk-yard" OR "junk yard" OR "log-lift" OR log-lift* OR "log lift" OR log lift* OR "log press" OR log press* OR "log-press" OR log-press* OR "yoke-walk" OR "yoke walk" OR "yoke-carry" OR "yoke carry" OR "super yoke" OR "super-yoke" OR "frame lift" OR frame lift* OR "frame-lift" OR frame-lift* OR "frame carry" OR "frame-carry" OR "farmers walk" OR "farmers carry" OR "farmer’s walk" OR "farmer’s carry" OR "suitcase carry" OR "duck walk" OR "frame carry" OR "hercules hold" OR "husafell stone" OR "tyre flip" OR tyre flip* OR "tyre-flip" OR tyre-flip* OR tyre lift* OR "tyre lift" OR "tyre-lift" OR tyre-lift* OR "tire-flip" OR tire-flip* OR "tire flip" OR tire flip* OR "tire lift" OR tire lift* OR "tire-lift" OR tire-lift* OR "car flip" OR car flip* OR "car-flip" OR car-flip* OR "atlas stone" OR atlas ston* OR "stone lift" OR stone lift* OR "conans wheel" OR "conan's wheel" OR "fingal’s fingers" OR "fingals fingers" OR "Vehicle pull" OR vehicle pull* OR "vehicle-pull" OR vehicle-pull* OR "sled pull" OR sled pull* OR "sled-pull" OR sled-pull* OR "sled tow" OR sled tow* OR "sled-tow" OR sled-tow* OR "truck pull" OR truck pull* OR "truck-pull" OR truck-pull* OR "car pull" OR car pull* OR "car-pull" OR car-pull* OR "chain drag" OR chain drag* OR "chain-drag" OR chain-drag* OR "rope drag" OR rope drag* OR "rope-drag" OR rope-drag* OR "sand bag" OR sand bag* OR "sand-bag" OR sand-bag* OR "sandbag" OR sandbag* OR "car lift" OR car lift* OR "car-lift" OR car-lift* OR "vehicle lift" OR vehicle lift* OR "vehicle-lift" OR vehicle-lift* OR "truck lift" OR truck lift* OR "truck-lift" OR truck-lift* OR "arm over arm pull" OR "arm-over-arm" OR "keg toss" OR "keg-toss" OR "axle press" OR axle press* OR "axle-press" OR axle-press* OR "dumbbell press" OR dumbbell press* OR "dumbbell-press" OR dumbbell-press*) **AND** ("biomechanic" OR biomechanic* OR "bio-mechanic" OR bio-mechanic* OR "kinetic" OR kinetic* OR "kinematic" OR kinematic* OR anthropomet* OR emg OR electromyograph* OR imu OR "inertial measurement unit" OR gait OR mechanic* OR force OR velocit* OR "force-velocity" OR time OR motion OR torque OR power OR "body mass" OR angular OR linear OR moment OR moment-angle OR "moment angle" OR moment-arm OR "moment arm" OR momentum OR displac* OR equilibrium OR acceler* OR reac* OR joint OR pressure OR inertia* OR work OR energy OR potential OR injur* OR impuls* OR 3D OR "motion capture") |
| --- | --- |
| **CINAHL** | (strongman OR "strong man" OR "strong-man" OR junkyard OR "junk-yard" OR "junk yard" OR "log-lift*" OR "log lift*" OR "log press*" OR "log-press*" OR "yoke-walk" OR "yoke walk" OR "yoke-carry" OR "yoke carry" OR "super yoke" OR "super-yoke" OR "frame lift*" OR "frame-lift*" OR "frame carry" OR "frame-carry" OR "farmers walk" OR "farmers carry" OR "farmer’s walk" OR "farmer’s carry" OR "suitcase carry" OR "duck walk" OR "frame carry" OR "hercules hold" OR "husafell stone" OR "tyre flip*" OR "tyre-flip*" OR "tyre lift*" OR "tyre-lift*" OR "tire-flip*" OR "tire flip*" OR "tire lift*" OR "tire-lift*" OR "car flip*" OR "car-flip*" OR "atlas ston*" OR "stone lift*" OR "conans wheel" OR "conan's wheel" OR "fingal’s fingers" OR "fingals fingers" OR "vehicle pull*" OR "vehicle-pull*" OR "sled pull*" OR "sled-pull*" OR "sled tow*" OR "sle-tow*" OR "truck pull*" OR "truck-pull*" OR "car pull*" OR "car-pull*" OR "chain drag*" OR "chain-drag*" OR "rope drag*" OR "rope-drag*" OR "sand bag*" OR "sand-bag*" OR "sandbag*" OR "car lift*" OR "car-lift*" OR "vehicle lift*" OR "vehicle-lift*" OR "truck lift*" OR "truck-lift*" OR "arm over arm pull" OR "arm-over-arm" OR "keg toss" OR "keg-toss" OR "axle press*" OR "axle-press*" OR "dumbbell press*" OR "dumbbell-press*") **AND** (biomechanic* OR "bio-mechanic*" OR (MH "Biomechanics+") OR kinetic* OR kinematic* OR anthropomet* OR emg OR electromyograph* OR imu OR "inertial measurement unit" OR gait OR mechanic* OR (MH "Mechanics+") OR force OR velocit* OR "force-velocity" OR time OR time OR (MH "Motion+") OR torque OR power OR "body mass" OR angular OR linear OR moment OR moment-angle OR "moment angle" OR moment-arm OR "moment arm" OR momentum OR displac* OR equilibrium OR acceler* OR reac* OR joint OR Pressure OR inertia* OR work OR energy OR potential OR injur* OR impuls* OR 3D OR "motion capture") |
| **Embase** | (strongman OR "strong man" OR "strong-man" OR junkyard OR "junk-yard" OR "junk yard" OR "log-lift*" OR "log lift*" OR "log press*" OR "log-press*" OR "yoke-walk" OR "yoke walk" OR "yoke-carry" OR "yoke carry" OR "super yoke" OR "super-yoke" OR "frame lift*" OR "frame-lift*" OR "frame carry" OR "frame-carry" OR "farmers walk" OR "farmers carry" OR "farmer s walk" OR "farmer s carry" OR "suitcase carry" OR "duck walk" OR "frame carry" OR "hercules hold" OR "husafell stone" OR "tyre flip*" OR "tyre-flip*" OR "tyre lift*" OR "tyre-lift*" OR "tire-flip*" OR "tire flip*" OR "tire lift*" OR "tire-lift*" OR "atlas ston*" OR "stone lift*" OR "conans wheel" OR "conan s wheel" OR "fingal s fingers" OR "fingals fingers" OR "car flip*" OR "car-flip*" OR "vehicle pull*" OR "vehicle-pull*" OR "sled pull*" OR "sled-pull*" OR "sled tow*" OR "sled-tow*" OR "truck pull*" OR "truck-pull*" OR "car pull*" OR "car-pull*" OR "chain drag*" OR "chain-drag*" OR "rope drag*" OR "rope-drag*" OR "sand bag*" OR "sand-bag*" OR "sandbag*" OR "car lift*" OR "car-lift*" OR "vehicle lift*" OR "vehicle-lift*" OR "truck lift*" OR "truck-lift*" OR "arm over arm pull" OR "arm-over-arm" OR "keg toss" OR "keg-toss" OR "axle press*" OR "axle-press*" OR "dumbbell press*" OR "dumbbell-press*") **AND** (biomechanic* OR "bio-mechanic*" OR kinetic* OR kinematic* OR anthropomet* OR emg OR electromyograph* OR imu OR "inertial measurement unit" OR gait OR mechanic* OR "force" OR velocit* OR "force-velocity" OR time OR motion OR torque OR power OR "body mass" OR angular OR linear OR moment OR "moment-angle" OR "moment angle" OR "moment-arm" OR "moment arm" OR momentum OR displac* OR equilibrium OR acceler* OR reac* OR joint OR pressure OR inertia* OR work OR energy OR potential OR injur* OR impuls* OR 3D OR "motion capture") |
| **Medline (Ovid)** | (strongman OR strong man.tw OR strong-man.tw OR junkyard OR junk-yard OR junk yard OR log-lift* OR log lift* OR log press* OR log-press* OR yoke-walk OR yoke walk OR yoke-carry OR yoke carry OR super yoke OR super-yoke OR frame lift* OR frame-lift* OR frame carry OR frame-carry OR farmers walk OR farmers carry OR farmer's walk OR farmer's carry OR suitcase carry OR duck walk OR frame carry OR hercules hold OR husafell stone OR tyre flip* OR tyre-flip* OR tyre lift* OR tyre-lift* OR tire-flip* OR tire flip* OR tire lift* OR tire-lift* OR car flip* OR car-flip* OR atlas ston* OR stone lift* OR conans wheel OR conan's wheel OR fingal's fingers OR fingals fingers OR vehicle pull* OR vehicle-pull* OR sled pull* OR sled-pull* OR sled tow* OR sled-tow* OR truck pull* OR truck-pull* OR car pull* OR car-pull* OR chain drag* OR chain-drag* OR rope drag* OR rope-drag* OR sand bag* OR sand-bag* OR sandbag* OR car lift* OR car-lift* OR vehicle lift* OR vehicle-lift* OR truck lift* OR truck-lift* OR arm over arm pull OR arm-over-arm OR keg toss OR keg-toss OR axle press* OR axle-press* OR dumbbell press* OR dumbbell-press*) **AND** (biomechanic* OR bio-mechanic* OR kinetic* OR kinematic* OR anthropomet* OR emg OR electromyograph* OR imu OR inertial measurement unit OR exp gait/ OR mechanic* OR force OR velocit* OR force-velocity OR time OR exp motion/ OR exp torque/ OR power OR body mass OR angular OR linear OR moment OR moment-angle OR moment angle OR moment-arm OR moment arm OR momentum OR displac* OR equilibrium OR acceler* OR reac* OR joint OR pressure OR inertia* OR work OR energy OR potential OR injur* OR impuls* OR 3D OR motion capture) |
| **SPORTDiscus** | (strongman OR DE "STRONG men" OR "strong man" OR "strong-man" OR junkyard OR "junk-yard" OR "junk yard" OR "log-lift*" OR "log lift*" OR "log press*" OR "log-press*" OR "yoke-walk" OR "yoke walk" OR "yoke-carry" OR "yoke carry" OR "super yoke" OR "super-yoke" OR "frame lift*" OR "frame-lift*" OR "frame carry" OR "frame-carry" OR "farmers walk" OR "farmers carry" OR "farmer's walk" OR "farmer’s carry" OR "suitcase carry" OR "duck walk" OR "frame carry" OR "hercules hold" OR "husafell stone" OR "tyre flip*" OR "tyre-flip*" OR "tyre lift*" OR "tyre-lift*" OR "tire-flip*" OR "tire flip*" OR "tire lift*" OR "tire-lift*" OR "car flip*" OR "car-flip*" OR "atlas ston*" OR "stone lift*" OR "conans wheel" OR "conan’s wheel" OR "fingal's fingers" OR "fingals fingers" OR "vehicle pull*" OR "vehicle-pull*" OR "sled pull*" OR "sled-pull*" OR "sled tow*" OR "sled-tow*" OR "truck pull*" OR "truck-pull*" OR "car pull*" OR "car-pull*" OR "chain drag*" OR "chain-drag*" OR "rope drag*" OR "rope-drag*" OR "sand bag*" OR "sand-bag*" OR "sandbag*" OR "car lift*" OR "car-lift*" OR "vehicle lift*" OR "vehicle-lift*" OR "truck lift*" OR "truck-lift*" OR "arm over arm pull" OR "arm-over-arm" OR "keg toss" OR "keg-toss" OR "axle press*" OR "axle-press*" OR "dumbbell press*" OR "dumbbell-press*") **AND** (biomechanic* OR "bio-mechanic*" OR kinetic* OR kinematic* OR DE "KINEMATICS" OR anthropomet* OR emg OR electromyograph* OR imu OR "inertial measurement unit" OR gait OR mechanic* OR force OR velcit* OR DE "SPEED" OR "force-velocity" OR time OR motion OR torque OR power OR "body mass" OR angular OR linear OR moment OR moment-angle OR "moment angle" OR moment-arm OR "moment arm" OR momentum OR displac* OR equilibrium OR acceler* OR reac* OR joint OR pressure OR inertia* OR work OR energy OR (DE "MUSCLE strength") OR potential OR injur* OR impuls* OR 3D OR "motion capture") |
